# ngs_backbone: a pipeline for read cleaning, mapping and SNP calling using Next Generation Sequence

**DOI:** 10.1186/1471-2164-12-285

**Published:** 2011-06-02

**Authors:** Jose M Blanca, Laura Pascual, Peio Ziarsolo, Fernando Nuez, Joaquin Cañizares

**Affiliations:** 1 Instituto de Conservación y Mejora de la Agrodiversidad Valenciana (COMAV), Universidad Politécnica de Valencia, Camino de Vera s/n, 46022 Valencia, Spain

## Abstract

**Background:**

The possibilities offered by next generation sequencing (NGS) platforms are revolutionizing biotechnological laboratories. Moreover, the combination of NGS sequencing and affordable high-throughput genotyping technologies is facilitating the rapid discovery and use of SNPs in non-model species. However, this abundance of sequences and polymorphisms creates new software needs. To fulfill these needs, we have developed a powerful, yet easy-to-use application.

**Results:**

The ngs_backbone software is a parallel pipeline capable of analyzing Sanger, 454, Illumina and SOLiD (Sequencing by Oligonucleotide Ligation and Detection) sequence reads. Its main supported analyses are: read cleaning, transcriptome assembly and annotation, read mapping and single nucleotide polymorphism (SNP) calling and selection. In order to build a truly useful tool, the software development was paired with a laboratory experiment. All public tomato Sanger EST reads plus 14.2 million Illumina reads were employed to test the tool and predict polymorphism in tomato. The cleaned reads were mapped to the SGN tomato transcriptome obtaining a coverage of 4.2 for Sanger and 8.5 for Illumina. 23,360 single nucleotide variations (SNVs) were predicted. A total of 76 SNVs were experimentally validated, and 85% were found to be real.

**Conclusions:**

ngs_backbone is a new software package capable of analyzing sequences produced by NGS technologies and predicting SNVs with great accuracy. In our tomato example, we created a highly polymorphic collection of SNVs that will be a useful resource for tomato researchers and breeders. The software developed along with its documentation is freely available under the AGPL license and can be downloaded from http://bioinf.comav.upv.es/ngs_backbone/ or http://github.com/JoseBlanca/franklin.

## Background

The possibilities offered by next-generation sequencing (NGS) platforms are revolutionizing biotechnological laboratories, but to fulfill their true potential one hurdle has yet to be overcome: data analysis [1]. Presently, getting a *de novo*, 454-based (454 Life Sciences, Roche. Branford, CT, USA[2]) sequence for a non-model species transcriptome or an Illumina-based (Illumina, San Diego, CA, USA[3]) genomic or transcriptomic resequencing of several samples is very affordable.

However, these new sequencing technologies cannot be analyzed with older software designed for Sanger sequencing. Both the quantity and quality of the data are very different [1,4]. A slew of new software and data formats are continually being created: assemblers (Mira [5], Newbler (454 Life Sciences, Roche. Branford, CT, USA[2]), mappers (e.g., bwa [6], Bowtie [7]) and file formats (SAMtools [8], VCF [9]). These fast-paced developments have made the field of bioinformatics very dynamic and therefore difficult to follow despite the guidance provided by resources like the SEQanswers internet forum [10], which is dedicated to presenting and documenting the tools used to analyze NGS data. In addition, once the optimal tools are selected and the analyses are finished, huge files are obtained. These files are usually processed by creating small pieces of software called scripts. In our opinion, both the selection of the various programs and parameters as well as the creation of these small scripts render the analysis process cumbersome and non-reproducible, especially if the laboratory lacks a dedicated bioinformatics staff.

These problems can be relieved by using a standardized method and easy-to-use pipelines capable of integrally analyzing all the steps required to go from raw NGS sequences to the set of final annotations. Some notable prior efforts in this area have produced several pipelines. Three prominent examples are Galaxy [11], CloVR [12] and est2assembly [13]. est2assembly is a good assembly pipeline, but unfortunately it is unable to process Illumina and SOLiD (Sequencing by Oligonucleotide Ligation and Detection) reads [14], does not work in parallel and does not do read mapping. The applications of the NGS sequencing platforms are endless [15-18]. For example, up until recently, the use of SNPs in non-model species has been uncommon. However, the combination of NGS sequencing and affordable, high-throughput genotyping technologies (like Massarray (Sequenom, San Diego, CA, USA), Veracode or BeadArray (Illumina, San Diego, CA, USA)[3] is facilitating the rapid discovery and use of these molecular markers. Millions of sequences can now be generated at a low cost, and, given an easy way to analyze them, a huge number of SNPs can be rapidly obtained. This abundance of SNPs creates a need for new software, as researchers are usually interested in selecting an SNP subset targeted at a specific experiment. The SNPs in these subsets should have a low error rate, should be adapted to the genotyping technique to be used and should be variable in the individuals to be genotyped.

We aimed to create a powerful yet easy-to-use application capable of performing NGS sequence analysis and polymorphism prediction. In order to build a truly useful tool, the software development was paired with a laboratory experiment: the search for SNVs in tomato. In this species, due to the narrow genetic base present [19-21], it has proven difficult to find highly polymorphic SNPs. Several previous studies have mined the public tomato EST databases [22-25]. In these studies, thousands of tomato SNPs were predicted between certain accessions. Unfortunately, the polymorphism for these SNPs in other tomato accessions has not been assessed nor reported, rendering these SNPs cumbersome to use in other tomato materials. Other approaches have been able to find tomato SNPs and report their polymorphism, but only a few hundred SNPs have been obtained by means of manual resequencing [26] and oligonucleotide array hybridization [27]. The search for novel and highly informative SNVs (single nucleotide variations, SNPs plus indels) by resequencing the tomato transcriptome will in and of itself be exceedingly useful for scientists and breeders working on this species.

The software developed along with its documentation is freely available under the AGPL license and can be downloaded from the COMAV's bioinformatics service web site [28], as well as in additional file 1.

## Implementation

When the architecture of ngs_backbone was created, several characteristics were regarded as important: the use of standard file formats and third-party free software tools, modularity and extensibility, analysis reproducibility and ease of use.

To facilitate interoperability with other tools, most input and output files have a standard format, such as FASTA, FASTQ, BAM, VCF and GFF, which can be produced and used by other tools. For instance, it is very easy to view the mapping and annotation obtained by loading the BAM and GFF files into a viewer, such as IGV [29].

ngs_backbone uses third-party tools of recognized quality, such as SAMtools or GATK whenever possible, in order to maintain the quality of the analyses. This approach takes its toll on the installation process, but in order to make it less complicated, we have packaged and precompiled most of these third party tools and have written a detailed step-by-step installation manual that is distributed with the tool [30].

Modularity was also an important design aim of the ngs_backbone architecture. Users demand an ever-changing set of analyses to be carried out, and these analyses have to be adjusted for every project. To meet this requirement, a collection of mapper functions focused on different tasks, such as cleaning or annotating, were created. These functions have a common interface, they all take a sequence and generate a new, modified sequence and constitute the steps of our pipelines, which are generated at runtime for every analysis.

Finally, even though we are presenting ngs_backbone as a command-line tool, this is not the only way to use it. The underlying library that powers this tool is called franklin and is written in Python. This library has other capabilities that at this time are not exposed through the present command line interface, but its API is documented and easy to use, and Python programmers willing to develop their own scripts and tools on top of it are welcome to do so. Its development can be followed at the github website [31] and its license is also open (AGPL).

## Results and Discussion

### ngs_backbone pipeline algorithms

This section describes the methods used internally by ngs_backbone. The third-party software cited is not supposed to be run by the user, as it will only be used internally by ngs_backbone. Only a couple of commands (backbone_create_project and backbone_analyze) will suffice to complete any analysis.

A typical analysis carried out by ngs_backbone starts with a set of Sanger, 454, Illumina or SOLiD read files. The first step is the read cleaning. In this process adaptor, vector and low-quality regions are removed. The exact algorithm used for every cleaning step depends on the type of read. For instance, quality trimming in the long reads is done by lucy [32], but for the shorter reads, an internally implemented algorithm is used instead. For more details about the host of read-cleaning modules available, refer to the documentation distributed with the tool [28]. Once the cleaning is finished, quality and length distributions can be created for the raw and clean reads as a quality assessment of the cleaning process.

If a reference transcriptome is unavailable, one can be assembled with the clean reads by using the MIRA assembler [5]. MIRA allows hybrid assemblies with Sanger, 454 and Illumina reads. ngs_backbone automates the preparation of a MIRA project. After running MIRA, the obtained set of contigs may be annotated with all available annotations: microsatellite, ORF, functional description, GO terms, intron location and orthologs.

Once a reference transcriptome or genome is available, the reads may be mapped onto it. For the mapping, the algorithm employed also depends on the read length. Short reads are mapped by the standard bwa [6] algorithm, while the longer reads use that of BWT-SW. ngs_backbone generates a BAM file with all the mapped reads in it. The generated BAM files are processed using SAMtools [8] and Picard [33], and are merged and adapted for GATK [34] running a custom code.

One frequent objective of the projects that use NGS sequences is to look for sequence variation (SNPs and small indels). To improve this process, a BAM file realignment may be done by using GATK [34] prior to the SNV calling. For the SNV calling, the reads with a mapping quality lower than 15 are not considered. The allele qualities are calculated by using the quality of the three most reliable reads (PQ1, PQ2, PQ2) using the formula PQ1 + 0.25 * (PQ2 + PQ3). This method is a slight variation of the one used by MIRA [5] to calculate the quality for a consensus position. The SNV annotation takes into account the accumulated sequence quality for every allele as well as the mapping quality for each read. A threshold is set for both parameters, and only positions with two or more high-quality alleles are considered as SNPs or indels. Thousands of SNVs are typically generated from these BAM files, so in order to be able to select the most useful ones, a set of SNV filters has been developed (Table 1). The code used to run the SNV filters was all custom code written for ngs_backbone. The SNVs finally obtained along with the filter information are stored in a VCF file [9].

**Table 1:** ngs_backbone filters for SNV selection.

	Description and pass conditions	Value
**MAF**	Frequency of most frequent allele in the selected pool allele is less than	0.80
**HVR**	Percentage of divergence in the unigene is smaller than or equal to	4
**UCR**	No duplicated or fragment regions are detected by Blast	--
**I30**	The distance from intron/exon boundary is greater than	30
**CL**	The distance to ends of unigene is greater than	30
**CS**	The distance from neighboring SNPs is greater than	60
**CEF**	The SNV can be detected by endonuclease restriction	--
**VK**	Select the kind of marker: SNP or indel	--
**GF**	Frequency of most frequent allele in the selected libraries is less than	0.67

Although the analysis explained is a typical one, each of the steps is in fact optional. The pipeline has several entry points and results. One could start with raw sequences and do just the cleaning, or alternatively start with the BAM file and use the tool to call the SNVs. Every analysis is independent of the others; it just takes a set of standard files as input and generates another set of standard files as output.

### Using the software, tomato ngs_backbone analysis

To test the tool, a complete analysis of the tomato transcriptome was carried out, from the read cleaning to the experimental SNV validation. All these analyses were done using ngs_backbone. All public tomato Sanger EST reads available at the SGN and GenBank databases [35,36] with known tomato accession origins were included in this study. In addition to these Sanger sequences, 14.2 million Illumina reads obtained from a normalized cDNA library, built with an equimolar mix of floral RNA extracted from the tomato lines UC-82 and RP75/79, were added (additional file 2).

**Figure 1:**
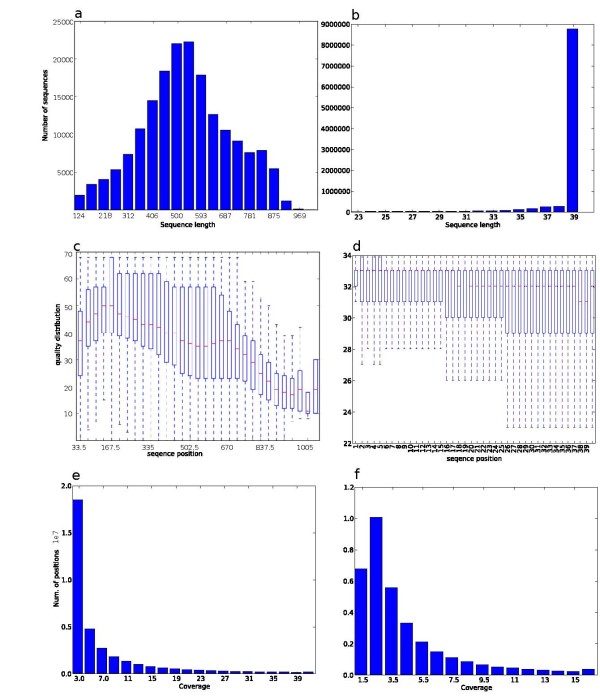
**ngs_backbone statistical analysis**. Sequence length distribution of cleaned Sanger (a) and Illumina (b) sequences. Boxplot of quality pair base lecture with respect to sequence position of Sanger (c) and Illumina (d) sequences. Alignment sequence coverage distribution of Sanger (e) and Illumina (f) sequences.

After removing the low-quality regions and vector and adaptor contaminants, 9.8 million Illumina and 276,039 Sanger sequences remained. The most-represented tomato lines were Micro Tom (118,304 sequences), TA496 (104,503 sequences) and the RP75/59-UC82 mix (9.8 million sequences). ngs_backbone calculated statistics about sequence features and the cleaning process (Figure 1).

The cleaned reads were mapped to the SGN tomato transcriptome [35]. 7.75 million Illumina as well as all Sanger reads were mapped, obtaining an average coverage of 4.2 for the Sanger and 8.5 for the Illumina sequences (Figure 1). To improve this alignment, the realignment functionality provided by GATK [34] was applied prior to the SNV calling.

The SNV annotation took into account the accumulated sequence quality for every allele as well as the mapping quality for each read. A threshold was set for both parameters, and only positions with two or more high-quality alleles were considered as SNPs or indels. All 33,306 SNVs found are reported in the VCF file (Additional File 3).

Despite satisfying the quality criteria, not all SNVs seemed equally reliable. Several filters were applied to tag those most likely to be real (Table 1). For example, a most frequent allele frequency (MAF) filter was applied to the Illumina set because a ratio between the alleles close to 0.5 is expected in most cases when two equimolar cDNA samples are mixed. In our case, the mix corresponded to the tomato lines UC-82 and RP75/79, and the alleles present in both of them were expected to appear in the ESTs an equal number of times for most unigenes. Also, a filter that labeled the SNVs in highly variable regions (HVR) was applied to avoid unigenes with too much variation. The 23,360 SNVs that passed both filters were considered to have a higher likelihood of being real and constituted the HL set (Table 2). The SNV counts presented from this point on will not include the SNVs that did not pass these filters unless explicitly stated.

**Table 2:** SNVs detected

	SA	IL	HL
**SNVs**	17237	19052	23306
**Indels**	3389	3044	5410
**SNPs**	13848	16008	27896
**SNVs_HVR4**	16575	17005	30827
**SNVs_MAF0.80**	-	9903	-
**SNVs_HVR4_MAF0.80**	16575	9640	23360

SA: Sanger collection: SNVs detected with Sanger sequences.

IL: Illumina collection: SNVs detected with Illumina sequences.

HL: Higher likelihood collection: SNVs detected with Illumina and Sanger sequences.

When using SNVs in an experimental setting, not all are equally useful and easy to use. Depending on the technique used to detect them, several SNV characteristics can ease or hinder an experiment, like closeness to an intron boundary, to another SNV or to the end of the unigene. Also, the SNVs located in unigenes that are very similar to other unigenes were tagged to avoid gene families that could make following the PCR and primer design processes difficult. This was done by applying the Unique and Continuous Region filters, I30 and CL30, available in the ngs_backbone filter collection (Table 1). All filters applied in order to label the SNVs as well as the results obtained are shown in Table 3. The 6,934 SNVs that passed these filters made up the easily usable set (EU).

**Table 3:** SNVs selected in the different collections using different ngs_backbone filters.

	SA	IL	HL	CO	PO
**SNVs**	16575	9640	23360	2855	514
**UCR**	11312	6763	16150	1925	294
**I30**	16502	9619	23271	2847	507
**CL30**	16249	8996	22523	2722	510
**EU**	4360	3434	6934	860	291
**CS60**	6155	4765	9730	1190	98
**CEF**	645	480	996	129	25

SA: Sanger collection: SNVs detected with Sanger sequences.

IL: Illumina collection: SNVs detected with Illumina sequences.

HL: Higher likelihood collection: SNVs detected with Illumina and Sanger sequences.

CO: Common collection: SNVs detected in Illumina and Sanger collections.

PO: Polymorphic collection: SNVs with an estimated frequency of most common allele under 0.67.

EU: Easily usable SNVs set: SNVs selected using UCR I30 and CL30 filters.

It is also desirable to tag the SNVs with high polymorphism.

The main advantage of these highly polymorphic markers consists in their ease of use across different individuals. SNVs with a low PIC (Polymorphic Information Content) have a low likelihood of having different alleles between two randomly chosen individuals. By enriching the selection with highly polymorphic SNVs, the proportion of discriminating SNVs in any experiment dealing with a random collection of individuals is increased, thereby reducing laboratory costs.

The polymorphism in a population can only be correctly inferred by having an extensive and well-genotyped sample of individuals. Since ESTs convey genotyping information from different individuals, ngs_backbone does a crude estimate of the polymorphism for each SNV by counting the number of tomato accessions in which each allele appears. The reliability of this inferred polymorphism depends on, among other parameters, the number of individuals sequenced. Taking into account only the SNVs sequenced in at least six different tomato accessions, the 514 SNVs with a frequency for the most common allele under 0.67 were included in the polymorphic (PO) set. This set was small in spite of the good sequence coverage for four of the tomato accessions, as not many sequences were available from other tomato materials. The intersection of this PO set with the easily usable one (EU) produced 291 SNVs.

To augment the number of putative highly polymorphic SNVs, less stringent criteria were also applied, creating a new set with the variable SNVs in both the Illumina and in the Sanger sequences, regardless of their estimated polymorphism. 2,855 SNVs were selected, of which 860 were also present in the EU selection (Table 3). These SNVs were denominated common (CO), as they were polymorphic in the public EST collection as well as in the Illumina sequences. The SNVs found only to be polymorphic in the Sanger or in the Illumina collections were named SA and IL, respectively.

### Experimental validation of software predictions

The quality of the *in silico *SNV calling was tested in a collection of 37 tomato accessions that included 10 commercial cultivars and 27 tomato landraces (Additional File 4). The technique used to genotype these materials was HRM PCR (High Resolution Melting PCR) [37]. To assign the melting curves to the SNV alleles, the accessions RP75/59 and UC-82, which comprise the Illumina EST set, were used as controls when possible. When no polymorphism was expected between these accessions, restriction enzyme polymorphism (also predicted by ngs_backbone) was used to differentiate the alleles.

A total of 76 *in silico *SNVs were experimentally tested (Additional Files 2, 5). The HRM technique was able to confirm 85% of these (Table 4). This high success rate makes the use of the *in silico*-predicted SNVs possible even without any previous extensive experimental validation. Moreover, the success rate was with all probability underestimated due to the experimental technique used. HRM PCR is not able to distinguish all allele pairs, and it is quite likely that in some cases the failure to detect some of the *in silico*-predicted SNVs was due to a flaw in the PCR.

**Table 4:** Statistics for assayed SNVs in the different collections.

	SNVs	HRM detected	% Polymorphic markers	Average frequency b	**PIC **c
**SA**	14	--	21.4	0.98	0.04
**IL**	14	12	41.7	0.95	0.09
**CO**	33	28	71.4	0.85	0.22
**PO**	15	13	69.2	0.80	0.28

**a **Percentage of polymorphic markers: number of polymorphic markers with respect to detected HRM markers or total markers for each set.

**b **Average frequency of most frequent allele of all detected markers or total markers for each set.

**c **PIC (polymorphic information index) of all detected markers or total markers for each set.

This high success rate was achieved despite the low coverage employed (4.2 for Sanger and 8.5 for Illumina), although it was probably obtained at the expense of a low specificity that was not assessed in the experimental design presented. The parameters used to do the selection were even adjusted so as to tag as unreliable some SNVs that, even though they were supported by enough coverage, were in regions with high variability or that presented an allele frequency that was off balance in the equimolar RP75/59 - UC-82 Illumina sample.

One of the aims of this study was to devise and test a strategy for selecting the most polymorphic SNV subset by using both the publicly available as well as the new Illumina ESTs. Although it is not possible to do an accurate PIC estimate just by using a collection of public sequences gathered from different heterogeneous projects, a rough index related to polymorphism might be calculated by counting the number of individuals in which each allele appears. Despite several confounding factors, a low PIC SNV will tend to produce very off-balance individual counts for the different alleles. The expected mean polymorphism of the SVN sets with different PICs was estimated by genotyping 37 tomato accessions. Two SNV sets were used to define the polymorphism baseline to expect. The only polymorphism-related filter applied to these sets was the requirement of having at least two different alleles in the Illumina or Sanger sequences. Once the tomato collection was genotyped, using SNVs randomly selected from these sets, we found that 3 out of 14 SNVs tested in the Sanger set and 5 out of 12 in the Illumina set were polymorphic, which is to say that the most frequent allele frequency was lower than 95%.

Other SNV sets that were expected to be somewhat more polymorphic were those built by sieving the SNVs that were polymorphic in both the Sanger and the Illumina sequences (CO set) as well as those from the PO set (where sequences from at least 6 plants were available and the allele count was quite balanced). In both sets, 70% of the markers tested were polymorphic, which was clearly higher than the 21% and 42% found in the polymorphism baseline.

In these sets, the polymorphic information content (PIC) was also expected to be higher than the one found in the Sanger and Illumina sets, where PIC was 0.04 and 0.08, respectively. In the CO set, the PIC was in fact higher, 0.22. Lastly, the SNVs that were expected to be most polymorphic were the ones from the PO set. In these, the sequences from at least 6 plants were available and the allele count was quite balanced. The PIC found, in this case, was 0.28, so when looking for highly polymorphic SNVs, this final strategy pays off.

Unfortunately, a selection like this cannot be done directly in all non-model species with public ESTs, as in many cases almost all sequences come from just a handful of different individuals. In fact, not even in public tomato sequences is there much diversity. 81% of these public sequences came from just 2 individuals. Given the results shown, we would recommend that, when looking for SNVs, the number of individuals sequenced be taken into account.

## Conclusion

To analyze NGS transcriptome data, we have developed a highly configurable, modular and parallel pipeline written in Python named ngs_backbone. This software presents a new strategy for using NGS technologies that will speed up research in non-model species and facilitate the use of these technologies by laboratories with or without a specialized bioinformatics staff.

In the tomato example presented, the analysis started with 14.5 million reads, which, after being cleaned and mapped, yielded 23,360 putative SNPs and indels (SNVs).

According to experimental validation, 85% of the *in silico*-predicted SNVs were real. This high success rate makes the use of the *in silico*-predicted SNVs possible even without any previous extensive experimental validation. In addition, the collection of 2,855 highly polymorphic SNVs created will be a useful resource as tomato landraces and vintages have a narrow genetic base, making it quite difficult to detect polymorphic markers in these materials.

The ngs_backbone software provides an ideal way to carry out a complete analysis on NGS sequences, including read cleaning, mapping, transcriptome assembly, annotation and SNV calling. This is an open-source tool released under the AGPL, written in Python and available at the COMAV's bioinformatics service web site [28] In addition, the underlying library that powers ngs-backbone is called franklin. Its development can be followed at the github website [31]. Its API is documented and easy to use, and Python programmers willing to develop their own scripts and tools on top of it are welcome to do so.

## Availability and requirements

**Project name: **ngs_backbone

**Project home page**: http://bioinf.comav.upv.es/ngs_backbone/ and http://github.com/JoseBlanca/franklin

**Operating system: **Linux

**Programing language: **Python (2.6)

**Other requirements: **Please refer to website for full listing.

**License: **Open source, AGPL

**Restrictions of use by non-academics: **none

## Authors' contributions

LP obtained the experimental data and participated in the analysis. JC designed the study and experiments and participated in the analysis. JMB and PZ developed the ngs-backbone software and participated in the analysis. FN selected and handled the plant material. JMB, LP, FN and JC wrote the manuscript. All authors read and approved the final manuscript.

## Supplementary Material

Additional file 1 **ngs_backbone 1.1.0 software**. ngs_backbone 1.1.0. Last version, released on 31-08-2010. Click here for file

Additional file 2 **Materials and methods**. materials and methods, for the experimental software validation. Click here for file

Additional file 3 **VCF data file containing all SNVs identified**. Data file containing all SNVs identified. The SNVs have not been selected with any additional filter, but the file includes the data for all ngs_backbone filters used in this work. Click here for file

Additional file 4 **Accessions employed in the SNV validation**. Landraces were provided by the COMAV genebank, comercial cultivars were obtained from Semilleros Cucala Agricola (Beniganim, Spain). Click here for file

Additional file 5 **Primer sequences**. File with primer sequences used in this work. Click here for file
