# Supplementary material for: ngs_backbone: a pipeline for read cleaning, mapping and SNP calling using Next Generation Sequence
Source: BMC Genomics. 2011 Jun 2;12:285. doi: 10.1186/1471-2164-12-285 (PMC3124440; doi:10.1186/1471-2164-12-285)
Supplement: Additional file 1 — ngs_backbone 1.1.0 software. ngs_backbone 1.1.0. Last version, released on 31-08-2010. [file 1471-2164-12-285-S1.GZ › ngs_backbone-1.1.0/doc/mapping.html]

Mapping — ngs\_backbone v0.1 documentation


# ngs\_backbone v0.1 documentation

index |
next |
previous

# Mapping¶

A set of read files can be mapped against a reference genome. For the mapping ngs\_backbone uses bwa with two algorithms, one for the long reads (sanger and 454) and other for the short reads (illumina). The result is a set of bam files one for each input read file or a merged bam file with all reads in it.

## Input and output files¶

The read files should be located in reads/cleaned/ and should follow the *naming conventions*. It is very important to set in the read file names the library, sample and platforms, otherwise the realignment and the SNP calling will fail. The reference genome should be located in mapping/reference as a fasta file.

Once bwa is finished a timestamped mapping directory will contain a bams/by\_readgroup subdirectory with one bam file for each input read file. Every one of such bam files is considered to be a read group. After the mapping is finished a merge\_bam analysis can be done. That analysis will merged all bam files located in bams/by\_reagroup and will create an unique bam file in bams/merged.bam. This bam file will contain as many read groups as bam files are merged. Every read group will retain the information about the library, sample and platform.

## Running the analysis¶

The analysis is run in divided in two ngs\_backbone analysis:

mapping
:   It maps the reads with bwa creating one bam for every input file

merge\_bam
:   It merges all bam files located in mapping/bams/by\_readgroup into mapping/bams/merged.bam. The obtained bam will comply not only with the samtools standard but also with the picard and GATK requirements.

# Bam realignment¶

This analysis does a GATK realignment. The mappings are usually done aligning each read with the reference genome at a time. These methodology can cause artifacts in the multiple sequence alignment obtained. GATK is capable of solving these artifacts. Their algorithm is described in its own site.

## Input and output files¶

The only one input file should be mapping/bams/merged.bam. This bam file contains all the reads mapped to the reference genome. The output file will be also mapping/bams/merged.bam (a versioned copy).

## Running the analysis¶

The corresponding ngs\_backbone is realign\_bam.

### Table Of Contents

- Introduction
- Usage
- Naming conventions
- Available analyses
- Parallel operation
- Installation
- Cleaning sequence reads
- Mira assembly
- Mapping
  - Input and output files
  - Running the analysis
- Bam realignment
- Annotation
- Snv filters
- Tutorials
- NGS workshop
- Licence
- Indices and tables
- seq\_io
- Architecture

### Search


Enter search terms or a module, class or function name.

index |
next |
previous
  
Show Source

© Copyright 2010, Jose Blanca.
Created using Sphinx 1.0pre.
