# Supplementary material for: ngs_backbone: a pipeline for read cleaning, mapping and SNP calling using Next Generation Sequence
Source: BMC Genomics. 2011 Jun 2;12:285. doi: 10.1186/1471-2164-12-285 (PMC3124440; doi:10.1186/1471-2164-12-285)
Supplement: Additional file 1 — ngs_backbone 1.1.0 software. ngs_backbone 1.1.0. Last version, released on 31-08-2010. [file 1471-2164-12-285-S1.GZ › ngs_backbone-1.1.0/doc/snv_filters.html]

Snv filters — ngs\_backbone v0.1 documentation


# ngs\_backbone v0.1 documentation

index |
next |
previous

# Snv filters¶

Once we have called the snvs with the annotate\_snv backbone analysis, we can filter those snvs.
This analysis is performed by the filter\_snv analysis.

The configuration of the snv filtering is handled by the snv\_filters section of the backbone.conf file. For each filter that we want to apply we have to create a new subsection like:

```
['snv_filters']
[['filter1']]
name = 'close_to_intron'
distance = 30
use  = True
```

All the snv filters have a boolean configuration parameter named *use*, when this parameter is set to False the filter will not be used even if it is declared in the configuration file.

By default only kind filtering is activated although, as an example, other filters are configured with its use parameter set to False. To use any of these filters change the *use* parameter to True in each of the filter sections:

```
use = True
```

None of the filters remove the snv, instead it adds a flag to the snv depending on the result of the filter. The information regarding the snv filtering will be written in the vcf file.

## Available filters¶

The configuration parameters for each available filter is described here.

### Close to intron¶

The snvs close to an intron will be filtered by this filter. To use it you have to annotate the introns before using the *cDNA intron annotation* backbone analysis.

Use this filter if you want to filter snvs that are closer than X nucleotides to an intron.
The configuration parameter for this filter is the distance between the snv and the intron:

```
name     = 'close_to_intron'
distance = 30
```

### Close to snv¶

Use this filter if you want to filter out snvs that are close to another snv.

This filter only have a parameter, distance between snvs:

```
name     = 'close_to_snv'
distance = 60
```

### Close to limit¶

Use this filter if you want to filter snvs that are close to the edges of the sequence.

The parameter to set for this filter is:

```
name     = 'close_to_limit'
distance = 60
```

### High variable region¶

It filters out all the snvs found in a sequence with a high variability. The variability is measured as the number of snvs per base.

Two parameters can be configured for this filter, the variability threshold and the window. If the window is not given the complete sequence is used to calculate the variability.

::
:   name = ‘high\_variable\_region’
    max\_variability = 0.04
    window = None #(All the sequence)

### More frequent allele¶

With this filter we can filter out the filters in which the most frequent allele has an allelic frequency above the given threshold. This filter can be used taking into account all the alleles or only the ones found in a subset of libraries, samples or readgroups.

To configure this filter with a threshold of 0.8 in a library named test\_library we would do:

```
maf        = 'maf'
frequency  = 0.8
group_kind = 'libraries'
groups     = ['test_library']
```

### Kind filter¶

The backbone snv caller annotates SNPs and indels, with this filter, for instance, we can filter out the snvs that are not SNPs.
The snv caller sets a kind for each snv: SNP, INDEL or COMPLEX. You can filter by:

```
'SNV', 'INVARIANT', 'COMPLEX'
```

This filter only has one option to configure:

```
name = 'by_kind'
kind = 'SNV'
```

### Cap enzymes¶

We can filter out the snvs that will not be detectable by using restriction enzymes. We consider a snv detectable if the two most abundant alleles have different restriction patterns.
This filter uses remap from EMBOSS to do the restriction mapping. We can choose to use all the restriction enzymes from the remap database or only a small subset of cheap and easily available enzymes. The parameter to configure is:

```
name        = 'cap_enzyme'
all_enzymes = True
```

### Sequence filter¶

It filters out the snv that do not belong to one of the given sequences. The sequences should be provided as a list in a file with one sequence name per line.

The filter requires a parameter with the path to the sequence names file:

```
name      = 'ref_not_in_list'
list_path = '/path/to/file/with/seq/names'
```

### Variable in group¶

With this filter we can select SNVs variables in some specific samples, libraries or read groups. For instance we can look for SNVs variables in the sample1.

It can work with libraries, samples or read\_groups. The kind of grouping used is set in the variable group\_kind.

The groups are a list (for instance a list of samples). By default it will look for SNVs variable in the union of these samples. If we ask for SNVs variable in sample\_1 and sample\_2 it will consider SNVs with different alleles between 1 and 2 and also SNVs with two alleles in sample\_1 but not variable in sample\_2. If we want all the samples to be variable independently of each other we could set the parameters in\_union to False and in\_all\_groups to True.

To configure the filter the kind of group to use (libraries, samples or read\_groups) should be set. Also a list with the group names should be given. A configuration to look for SNVs variable in the libraries lib1 and lib2 would be:

```
name        = 'is_variable'
unique_name = 'is_variable_in_lb'
group_kind  = 'libraries'
groups      = ['lib1', 'lib2']
```

Several filters of this kind can be set up in the configuration file, to distinguish them a unique\_name option with a should be also set up in the configuration for each filter.

### Not variable in group¶

With this filter we can select SNVs not variable in some specific samples, libraries or read groups. For instance we can look for SNVs not variables in the sample\_1.

It can work with libraries, samples or read\_groups. The kind of grouping used is set in the variable group\_kind.

The groups are a list (for instance a list of samples). If we ask for SNVs not variable in sample\_1 and sample\_2 it will consider SNVs that are not variable in all samples (they all should have the same allele). If we want the samples to be not variable but we want to allow to have different alleles between them we could use the option in\_union=False.

To configure the filter the kind of group to use (libraries, samples or read\_groups) should be set. Also a list with the group names should be given. A configuration to look for SNVs not variable in the libraries lib1 and lib2 would be:

```
name        = 'is_not_variable'
unique_name = 'not_is_variable_in_lb'
group_kind  = 'libraries'
groups      = ['lib1', 'lib2']
```

Several filters of this kind can be set up in the configuration file, to distinguish them a unique\_name option with a should be also set up in the configuration for each filter.

### Unique contiguous¶

With this filter you can filter out snvs that are in regions that seem to be duplicated or that are not contiguous.

This filter have 4 configurable options:

```
name               = 'uniq_contiguous'
distance           = 'distance from each side of the snv to select a region'
genomic_db         = '/path/to/the/seq/fasta/file'
genomic_seqs_fpath = '/path/to/the/seq/blast/db'
```

### Minimun number of groups¶

It filters out the SNVs that not supported by enough number of groups. For instance we could filter the SNVs read in less than 4 samples.

The options are:

```
name       = 'min_groups'
min_groups = 4
group_kind = 'samples'
```

### Table Of Contents

- Introduction
- Usage
- Naming conventions
- Available analyses
- Parallel operation
- Installation
- Cleaning sequence reads
- Mira assembly
- Mapping
- Bam realignment
- Annotation
- Snv filters
  - Available filters
- Tutorials
- NGS workshop
- Licence
- Indices and tables
- seq\_io
- Architecture

### Search


Enter search terms or a module, class or function name.

index |
next |
previous
  
Show Source

© Copyright 2010, Jose Blanca.
Created using Sphinx 1.0pre.
