# Supplementary material for: ngs_backbone: a pipeline for read cleaning, mapping and SNP calling using Next Generation Sequence
Source: BMC Genomics. 2011 Jun 2;12:285. doi: 10.1186/1471-2164-12-285 (PMC3124440; doi:10.1186/1471-2164-12-285)
Supplement: Additional file 1 — ngs_backbone 1.1.0 software. ngs_backbone 1.1.0. Last version, released on 31-08-2010. [file 1471-2164-12-285-S1.GZ › ngs_backbone-1.1.0/doc/install.html]

Installation — ngs\_backbone v0.1 documentation


# ngs\_backbone v0.1 documentation

index |
next |
previous

# Installation¶

This is a summary on how to install ngs\_backbone, below you have a *detailed explanation*.

To install ngs\_backbone python 2.6 is required. Also you need the python libraries Biopython, and ConfigObj. Other optional, but highly recommended, dependencies are: psubprocess, pysam and matplotlib. python is installed by default in the usual Linux distributions but you should check the version. Installing a python library is as easy as installing ngs\_backbone. Once you have the tarball downloaded just run the following command:

```
$ python2.6 setup.py install
```

ngs\_backbone requires also several external tools to run the analyses. You don’t need all of them for every analysis, depending on the analysis that you want to do you will require some subset of these tools. If you happen to run a x64 linux box installing the majority of these tools is a breeze, just download the x64 3erd party tools bundle and copy them to somewhere in your path. If you want to compile them get the source.

| analysis | external tools required |
| --- | --- |
| *Cleaning sequence reads* | lucy, exonerate, blast, Univec (database), mdust, trimpoly |
| *Mira assembly* | mira |
| *Mapping* | bwa, samtools, picard |
| *Bam realignment* | GATK |
| *SNP calling* | pysam |
| *ORF annotation* | ESTScan |
| *Ortholog annotation* | blast |
| *Description annotation* | blast |
| *Microsatellite annotation* | sputnik |
| *cDNA intron annotation* | blast, emboss |
| *GO annotation* | blast, blast2go |

## Step by step installation instructions¶

### python tools¶

ngs\_backbone requires python2.6. If you don’t have it already installed in your distribution download the source code and install it. The other requirements are python libraries. Biopython and ConfigObj are required and pysam, psubprocess and matplotlib are optional.

If your distribution include python2.6 chances are that Biopython and ConfigObj might be packages by your distribution, but we are going to explain here the manual process. The install process is simple, you just have to download a bunch of python tools, unpack them and run “python2.6 install” on them.

To install Biopython you need Numpy. Download Biopython and install it.

::
:   $ tar -xvzf numpy-1.4.1.tar.gz
    $ cd numpy-1.4.1

    $ tar -xvzf biopython-1.54.tar.gz
    $ cd biopython-1.54
    $ python2.6 setup.py install

Download ConfigObj and install it.

```
$ unzip configobj-4.7.1.zip
$ cd configobj-4.7.1
$ python2.6 setup.py install
```

Download and install psubprocess (If you don’t install it you won’t be able to run ngs\_backbone in parallel).

```
$ tar -xvzf psubprocess.0.1.1.tar.gz
$ cd psubprocess.0.1.1
$ python2.6 setup.py install
```

To call the SNP you will need the library pysam. pysam requires pyrex, so install it.

```
$ tar -xvzf Pyrex-0.9.9.tar.gz
$ cd Pyrex-0.9.9
$ python2.6 setup.py install
$ tar -xvzf pysam-0.2.tar.gz
$ cd pysam-0.2
$ python2.6 setup.py install
```

To create the charts for the statistics you will need matplotlib.

```
$ tar -xvzf matplotlib-0.99.3.tar.gz
$ cd matplotlib-0.99.3
$ python2.6 setup.py install
```

Once we have it all we can install ngs\_backbone.

```
$ tar -xvzf ngs_backbone.0.2.0.tar.gz
$ cd ngs_backbone.0.2.0
$ python2.6 setup.py install
```

### C tools¶

Most of the tools required has been precompiled for the x64 linux systems, download them and copy them to somewhere in your path.

```
$ tar -xvzf backbone_3er_party.tar.gz
$ cp -r backbone_3er_party_bin/* /usr/local/bin
```

Two other requirements are blast and emboss, chances are that you can install them using your distribution package manager. In Debian we would do:

```
$ apt-get install emboss
$ apt-get install blast2
```

blast without databases is of no use, as an example we are going to install the Univec database. After downloading the fasta file we uncompress it in a directory and we format it.

```
$ mkdir /srv/blast/
$ mv UniVec /srv/blast
$ formatdb -i UniVec -V -p F -o
```

If you want to do an assembly you will also need mira.

```
$ tar -xvjf mira_3.0.5_prod_linux-gnu_x86_64_static.tar.bz2
$ cp mira_3.0.5_prod_linux-gnu_x86_64_static/bin/mira /usr/local/bin
```

### Java tools¶

Two java tools are used to manage the sam files: picard and GATK. They are java tools, so you have to install java in your linux box.

```
$ apt-get install sun-java6-jre
```

blast2go, picard and GATK are easy to install, just download them and unpack them.

```
$ unzip picard-tools-1.22.zip
$ mv picard-tools-1.22 /usr/local
$ tar -xvjf GenomeAnalysisTK-latest.tar.bz2
$ mv GenomeAnalysisTK-1.0.3471 /usr/local
$ updatedb
```

It is advisable to run updatedb after setting everything to ease the ngs\_backbone configuration.

After installing the whole pipeline you can run the *NGS workshop tutorial* to test the whole system.

### Table Of Contents

- Introduction
- Usage
- Naming conventions
- Available analyses
- Parallel operation
- Installation
  - Step by step installation instructions
- Cleaning sequence reads
- Mira assembly
- Mapping
- Bam realignment
- Annotation
- Snv filters
- Tutorials
- NGS workshop
- Licence
- Indices and tables
- seq\_io
- Architecture

### Search


Enter search terms or a module, class or function name.

index |
next |
previous
  
Show Source

© Copyright 2010, Jose Blanca.
Created using Sphinx 1.0pre.
