# Supplementary material for: ngs_backbone: a pipeline for read cleaning, mapping and SNP calling using Next Generation Sequence
Source: BMC Genomics. 2011 Jun 2;12:285. doi: 10.1186/1471-2164-12-285 (PMC3124440; doi:10.1186/1471-2164-12-285)
Supplement: Additional file 1 — ngs_backbone 1.1.0 software. ngs_backbone 1.1.0. Last version, released on 31-08-2010. [file 1471-2164-12-285-S1.GZ › ngs_backbone-1.1.0/doc/index.html]

ngs\_backbone — ngs\_backbone v0.1 documentation


# ngs\_backbone v0.1 documentation

index |
next

# ngs\_backbone¶

ngs\_backbone is a bioinformatic application created to work on sequence analysis by using NGS (Next Generation Sequencing) and sanger sequences. It is capable of cleaning reads, do de novo assembly or mapping against a reference and annotate SNPs, SSRs, ORFs, GO terms and sequence descriptions.
Our laboratory is focused on transcriptomic analysis, so the tool has been used and tested on transcriptomes. Some analyses will be useful for genome analysis, but since our work deals mainly with transcriptomes design tradeoffs in ngs\_backbone reflect this.

ngs\_backbone can run in parallel using single node multicore systems and computer clusters.

For the analyses, in most cases, ngs\_backbone uses external software like: mira, bwa, samtools, picard, etc.

The application works on Linux so experience with that operating system is required when using it.

ngs\_backbone is free software so contributions and shared development will be welcomed.

If you find any problem when running ngs\_backbone please inform us.

### Table Of Contents

- Introduction
- Usage
- Naming conventions
- Available analyses
- Parallel operation
- Installation
- Cleaning sequence reads
- Mira assembly
- Mapping
- Bam realignment
- Annotation
- Snv filters
- Tutorials
- NGS workshop
- Licence
- Indices and tables
- seq\_io
- Architecture

### Search


Enter search terms or a module, class or function name.

index |
next
  
Show Source

© Copyright 2010, Jose Blanca.
Created using Sphinx 1.0pre.
