# Supplementary material for: ngs_backbone: a pipeline for read cleaning, mapping and SNP calling using Next Generation Sequence
Source: BMC Genomics. 2011 Jun 2;12:285. doi: 10.1186/1471-2164-12-285 (PMC3124440; doi:10.1186/1471-2164-12-285)
Supplement: Additional file 1 — ngs_backbone 1.1.0 software. ngs_backbone 1.1.0. Last version, released on 31-08-2010. [file 1471-2164-12-285-S1.GZ › ngs_backbone-1.1.0/doc/search.html]

Search — ngs\_backbone v0.1 documentation


# ngs\_backbone v0.1 documentation

index

# Search

Please activate JavaScript to enable the search
functionality.

From here you can search these documents. Enter your search
words into the box below and click "search". Note that the search
function will automatically search for all of the words. Pages
containing fewer words won't appear in the result list.

### Table Of Contents

- Introduction
- Usage
- Naming conventions
- Available analyses
- Parallel operation
- Installation
- Cleaning sequence reads
- Mira assembly
- Mapping
- Bam realignment
- Annotation
- Snv filters
- Tutorials
- NGS workshop
- Licence
- Indices and tables
- seq\_io
- Architecture

### Search


Enter search terms or a module, class or function name.

index

© Copyright 2010, Jose Blanca.
Created using Sphinx 1.0pre.
