# Supplementary material for: ngs_backbone: a pipeline for read cleaning, mapping and SNP calling using Next Generation Sequence
Source: BMC Genomics. 2011 Jun 2;12:285. doi: 10.1186/1471-2164-12-285 (PMC3124440; doi:10.1186/1471-2164-12-285)
Supplement: Additional file 1 — ngs_backbone 1.1.0 software. ngs_backbone 1.1.0. Last version, released on 31-08-2010. [file 1471-2164-12-285-S1.GZ › ngs_backbone-1.1.0/doc/assembly.html]

Mira assembly — ngs\_backbone v0.1 documentation


# ngs\_backbone v0.1 documentation

index |
next |
previous

# Mira assembly¶

The mira assembler is used to create a set of contigs with the sequence reads. The reads can come from 454, sanger and illumina sequencing. Hybrid assembly are possible. For mira configuration details refer to its documentation.

## Input and output files¶

The input files required to do a mira analysis are the reads located in reads/cleaned. The reads files should follow the *naming conventions*.

## Configuration parameters¶

The default configuration is tailored to EST assemblies. To modify the mira command line parameters you should go to the mira section in the ngs\_backbone.conf file. The options are:

job\_options
:   The mira job options parameter. By default they are: denovo, est

general settings
:   The parameters that affect all platforms.

454\_settings
:   The parameters that affect the 454 reads.

sanger\_settings
:   The parameters that affect the sanger reads.

## Running the analysis¶

The mira assembly analysis is divided into three ngs\_backbone analyses: prepare\_mira\_assembly, mira\_assembly and select\_last\_assembly.

The analysis prepare\_mira\_assembly will create the files required as input by mira in the directory assembly/input/. These files will be created taking the reads from reads/cleaned/.

The mira\_assembly analysis runs mira and creates the contigs. The files created by this analysis will be located at a timestamped directory located in assembly/. Several assemblies could be created with different parameters and each one would go into a different timestamped directory. Inside these directories a result subdirectory is created with the relevant result files.

The select\_last\_assembly will just make a soft link named assembly/result that points to the result subdirectory located in the latest timestamped assembly.

### Table Of Contents

- Introduction
- Usage
- Naming conventions
- Available analyses
- Parallel operation
- Installation
- Cleaning sequence reads
- Mira assembly
  - Input and output files
  - Configuration parameters
  - Running the analysis
- Mapping
- Bam realignment
- Annotation
- Snv filters
- Tutorials
- NGS workshop
- Licence
- Indices and tables
- seq\_io
- Architecture

### Search


Enter search terms or a module, class or function name.

index |
next |
previous
  
Show Source

© Copyright 2010, Jose Blanca.
Created using Sphinx 1.0pre.
