# Supplementary material for: ngs_backbone: a pipeline for read cleaning, mapping and SNP calling using Next Generation Sequence
Source: BMC Genomics. 2011 Jun 2;12:285. doi: 10.1186/1471-2164-12-285 (PMC3124440; doi:10.1186/1471-2164-12-285)
Supplement: Additional file 1 — ngs_backbone 1.1.0 software. ngs_backbone 1.1.0. Last version, released on 31-08-2010. [file 1471-2164-12-285-S1.GZ › ngs_backbone-1.1.0/doc/ngs_workshop/mapping.html]

Mapping — ngs\_backbone v0.1 documentation


# ngs\_backbone v0.1 documentation

index |
next |
previous

# Mapping¶

The mapping is the process of comparing each one of the reads with the reference genome. We will obtain an alignment between each read and the genome. Like for any other bioinformatic task there is a lot of mapping software available. The most commonly used programs are bowtie and bwa. These tools differ on the algorithm used, the sensitivity, the memory requirements, the speed, and the sequence length requirements.

Before choosing one it would be wise to read its manual pages. There are subtleties regarding how they deal (or not) with the insertions and deletions and with the repeated regions.

# sam format¶

In general all mappers render the result in a common file format, the sam format. This format is not meant for human consumption, although we can open the text version of the file. There is a growing collection of software created for dealing with these files. We can merge, sort, filter, realign and browse them. Some useful programs are:

> - samtools and picard to merge, sort and filter.
> - IGV for browsing.
> - GATK to recalibrate the quality scores and to realign the sequences.

Also the most common SNP callers would require a sam file to work.

We can encounter sam files in two flavors: sam and bam. The bam is the binary version and the sam is just the equivalent text file. The hold exactly the same information and we can convert between them with the samtools.

# sam realignment¶

The mapping is done read by read, so in some cases the multiple alignment could be not perfect. The GATK software has an option to realign a bam file generating a new one with this problem solved. It would be advisable running this analysis, specially if we are going to take into account the small indels.

# Task 3: read mapping¶

We want to map the reads found in the directory reads/cleaned to the maize transcriptome. To do it ngs\_backbone needs

Once we have cleaned the sequences it would be nice to get statistics about the raw and the cleaned sequences. But before doing this analysis we will add the reads that were cleaned in the original data. At any step ngs\_backbone will accept new files as long as the naming conventions are followed. So we add the cleaned that were previously cleaned to the reads/cleaned/ directory:

```
~/ngs_workshop/maize$ cp ../course_data/sm_Mo17.lb_Mo17_root.pl_454.sfastq reads/cleaned/
~/ngs_workshop/maize$ ls reads/cleaned/
sm_B73XMo17.lb_MB_14day.pl_illumina.sfastq  sm_Mo17.lb_Mo17_root.pl_454.sfastq
sm_mix.lb_genbank.pl_sanger.fasta           stats
```

To do the mapping we also require a reference sequence. In this case we will use some unigenes from the maize transcriptome. This reference should be located in mapping/reference/reference.fasta, so we create the directories and copy the files:

```
~/ngs_workshop/maize$ mkdir mapping
~/ngs_workshop/maize$ mkdir mapping/reference
~/ngs_workshop/maize$ cp ~/ngs_workshop/course_data/maize_unigenes.fasta mapping/reference/reference.fasta
```

Now that we have the cleaned reads and the reference we can do the mapping:

```
~/ngs_workshop/maize$ backbone_analysis.py -a mapping
2010-05-21 10:00:14,781 INFO MappingAnalyzer
2010-05-21 10:00:14,781 INFO backbone version: 0.1.0
2010-05-21 10:00:14,781 INFO Analysis started
2010-05-21 10:07:15,915 INFO Analysis finished
2010-05-21 10:07:15,915 INFO Time elapsed 0:07:01.176088
```

Once the analysis is completed we will find a timestamped directory in mapping. We could carry out another mapping with different parameters and another timestamped directory would be created. A link to this directory named bams is generated. In the mapping/bams/by\_readgroups/ we will have one bam file for every input cleaned read file.

It is very important that this bam files have the lb, sm and pl keys in the name, otherwise the next analysis would fail. ngs\_backbone generates the names for these files from the file names found in the reads/cleaned.

It is quite convenient for some analysis to have all mappings in one bam file. We can merge the bam that we have into a single one. This bam will retain the information about which read group every read belongs. To merge the bam we do:

```
~/ngs_workshop/maize$ backbone_analysis.py -a merge_bams
2010-05-21 10:10:40,723 INFO MergeBamAnalyzer
2010-05-21 10:10:40,723 INFO backbone version: 0.1.0
2010-05-21 10:10:40,724 INFO Analysis started
2010-05-21 10:13:28,457 INFO Analysis finished
2010-05-21 10:13:28,588 INFO Time elapsed 0:02:47.905998
```

Now we have a new merged.bam in mapping/bams/merged.0.bam. This file has all the mapped reads.

```
~/ngs_workshop/maize$ ls mapping/bams
by_readgroup  merged.0.bam  merged.0.bam.bai
```

# Task 3: Taking a look at a bam file¶

The bam is a binary file, but we could transform it into a text file using the samtools. However this is not a good way to take a look at it. There are some align viewers capable of opening the bam file, one of the best is the Integrative Genome Viewer (IGV). IGV has an extensive User Guide, so if you have any doubts refer to it.

Before loading our bam file we have to import our reference genome into IGV: File -> Import Genome.

Now we can load the mapping into IGV: File -> Load from file and load the merged bam file.

We would see something like:

Play around with IGV, select different unigenes, zoom into a region, look for some SNP.

### Table Of Contents

- Introduction
- Usage
- Naming conventions
- Available analyses
- Parallel operation
- Installation
- Cleaning sequence reads
- Mira assembly
- Mapping
- Bam realignment
- Annotation
- Snv filters
- Tutorials
- NGS workshop
  - Next Generation Sequencing
  - Platforms
  - Software
  - File formats
  - Read Cleaning
  - Task 1: cleaning the reads
  - Task 2: read statistics
  - Assembly vs mapping
  - Mapping
  - sam format
  - sam realignment
  - Task 3: read mapping
  - Task 3: Taking a look at a bam file
  - SNP calling
  - SNP filtering
  - VCF format
  - GFF format
  - Task 4: SNP calling
  - Task 5: Looking at the SNPs using IGV
  - Task 5: SNP filtering
  - Command line primer
- Licence
- Indices and tables
- seq\_io
- Architecture

### Search


Enter search terms or a module, class or function name.

index |
next |
previous
  
Show Source

© Copyright 2010, Jose Blanca.
Created using Sphinx 1.0pre.
