# Supplementary material for: ngs_backbone: a pipeline for read cleaning, mapping and SNP calling using Next Generation Sequence
Source: BMC Genomics. 2011 Jun 2;12:285. doi: 10.1186/1471-2164-12-285 (PMC3124440; doi:10.1186/1471-2164-12-285)
Supplement: Additional file 1 — ngs_backbone 1.1.0 software. ngs_backbone 1.1.0. Last version, released on 31-08-2010. [file 1471-2164-12-285-S1.GZ › ngs_backbone-1.1.0/doc/ngs_workshop/cmd_primer.html]

Command line primer — ngs\_backbone v0.1 documentation


# ngs\_backbone v0.1 documentation

index |
next |
previous

# Command line primer¶

To use ngs\_backbone you have to use the linux command line, but if you don’t have any previous command line experience don’t worry, it won’t be difficult.

We can tell a computer what to do using the mouse and pointing and clicking at buttons and menus or we just can tell them. For instance if we want to get a list of the files in a folder we can can open a folder browser and click our way to the folder or we can just write:

```
$ ls folder
file_1.txt
file_2.txt
```

None of these two ways of communicating with the computer is better. Some tasks are more easy to do with the mouse and some others writing with the keyboard, although some say that real men use command line.

## Into the real¶

To use the command line launch a terminal. You will see something like:

```
user_name $ _
```

We can write any command into this prompt and wait for the result. For instance we can tell the computer to list the files. Just write “ls” and press the enter key:

```
user_name $ ls
some_file.txt
another_file.txt
```

The ls command is in fact a fully fledged program. When we run a command we are executing a program that returns an output (in this case in the form of a text output) and it some cases it might also take an input. When we use the command line we run a command (call a program), we wait for the program to finish and we run another program.

Usually the commands modify its behaviour by using parameters. For instance we can tell ls to use the long format to print the file by writing:

```
$ ls -l
```

Every command has a collection of parameters and options. The standard way to ask for help about them is to use the option ‘–help’ or ‘-h’:

```
$ ls --help
Modo de empleo: ls [OPCIÓN]... [FICHERO]...
List information about the FILEs (the current directory by default).
Sort entries alphabetically if none of -cftuvSUX nor --sort.
```

## File system¶

The files in the computer are organized in folders and those folders are sorted in an hierarchical structure. When the program that we are executing requires a file or a folder as input we have to give this program the path to this input. We could do:

```
$ ls my_documents/
```

This time the ls command would list the contents in my\_documents. But what is ls listing when we invoke it without any parameter? There is a default directory that the ls command take. This directory is called the working directory and it is an important concept in a command line session. We are always inside a directory and this directory is called working directory. We can print this directory with the command:

```
$ pwd
/home/jose
```

We can change this directory using the command cd (Change Directory):

```
$ pwd
/home/jose
$ ls
dir_1
file_1.txt
$ ls dir_1
file_2.txt
$ cd dir_1
$ ls
file_2.txt
```

If you want your life to be easier do not use spaces or non-english alphanumerical characters in the file name. They can be used, but some programs might have problems with them.

## Directories¶

In a unix system like Linux or MacOS X the directory paths use forward slashes “/”. / is the root directory and /home/ is a subdirectory of /.

Every user has a “home” directory. In this directory the user can write and delete files and directories. In a Linux system this directory is usually /home/user\_name.

## Moving around the file system¶

```
pwd               print working directory, or current directory.
cd my_docs        Change current directory to the subdirectory my_docs.
cd                Change current directory to your HOME directory.
cd ..             Change current directory to the parent directory of the current directory.
```

## Listing directory contents¶

```
ls       list a directory
ls -l    list a directory in long ( detailed ) format
```

## Moving, renaming, and copying files¶

```
cp file1 file2          copy the file1 to file2
mv file1 newname        move or rename a file
mv file1 AAA/           move file1 into sub-directory AAA
rm file1                remove (delete) a file
rm -r dir1              recursively remove a directory and its contents
mkdir dir1              create a directory
```

## Viewing and editing files¶

The unix systems have powerful text tools. We can print, browse, filter, sort, cut text files easily. It is highly recommended to learn to use this commands if we deal with long text files.

```
cat filename            Dump a file to the screen.
less filename           Browse through a file: q=quit, / search.
head filename           Show the first lines of a file.
head -n  filename       Show the first n lines of a file.
tail filename           Show the last few lines of a file.
tail -n filename        Show the last n lines of a file.
grep string filename    prints all the lines in a file that contain the string
cut filename            cut some columns
```

## Interactive History¶

In the prompt we can use the arrow keys to access our previous commands, edit them and re-execute them.

### Table Of Contents

- Introduction
- Usage
- Naming conventions
- Available analyses
- Parallel operation
- Installation
- Cleaning sequence reads
- Mira assembly
- Mapping
- Bam realignment
- Annotation
- Snv filters
- Tutorials
- NGS workshop
  - Next Generation Sequencing
  - Platforms
  - Software
  - File formats
  - Read Cleaning
  - Task 1: cleaning the reads
  - Task 2: read statistics
  - Assembly vs mapping
  - Mapping
  - sam format
  - sam realignment
  - Task 3: read mapping
  - Task 3: Taking a look at a bam file
  - SNP calling
  - SNP filtering
  - VCF format
  - GFF format
  - Task 4: SNP calling
  - Task 5: Looking at the SNPs using IGV
  - Task 5: SNP filtering
  - Command line primer
- Licence
- Indices and tables
- seq\_io
- Architecture

### Search


Enter search terms or a module, class or function name.

index |
next |
previous
  
Show Source

© Copyright 2010, Jose Blanca.
Created using Sphinx 1.0pre.
