# Supplementary material for: ngs_backbone: a pipeline for read cleaning, mapping and SNP calling using Next Generation Sequence
Source: BMC Genomics. 2011 Jun 2;12:285. doi: 10.1186/1471-2164-12-285 (PMC3124440; doi:10.1186/1471-2164-12-285)
Supplement: Additional file 1 — ngs_backbone 1.1.0 software. ngs_backbone 1.1.0. Last version, released on 31-08-2010. [file 1471-2164-12-285-S1.GZ › ngs_backbone-1.1.0/doc/ngs_workshop/ngs.html]

Next Generation Sequencing — ngs\_backbone v0.1 documentation


# ngs\_backbone v0.1 documentation

index |
next |
previous

# Next Generation Sequencing¶

The new sequencing technologies characterized by parallel sequencing and high throughput are collective called Next Generation Sequence technologies. The main difference between the classic sanger sequencing and the new approaches is the massive parallelization.

The modern sanger sequencing is based on a capillary electrophoresis, every sequencing reaction is processed by using an electrophoresis. Although there are sanger sequencing machines capable of doing up to 384 parallel electrophoresis it is very difficult to augment more the number of sequencing reactions done in parallel. The most important breakthrough of the new sequencing technologies is the surface sequencing. In all of them the sequences are build by looking at a spot in a surface. Millions of spots can be individually distinguished in a slice, thus millions of sequences can be generated in parallel.

Previously we could get 1000 sanger reads for 3000$ now we can get the whole A. thaliana genome with a 13x coverage for this price. The cost per megabase has gone from 4000$ to 1.3$. Right now any small laboratory has the sequencing power that five years ago only the great sequecing centers had.

# Platforms¶

There are several sequencing commercially available NGS platforms: 454, illumina and Solid. Several good reviews are available, like:

> - Next-generation DNA sequencing
> - Sequencing technologies - the next generation

These platforms differ in the number of sequences generated and in the length and qualities of these sequences.

| Vendor | Roche | Illumina | ABI |
| --- | --- | --- | --- |
| Platform | 454 Ti | Solexa GA IIx | SOLiD 2 |
| Reads (Millions) | 1.25 | 250 | 115 |
| Read length | 400 | 100 | 35 |
| Yield (Gigabases) | 0.5 | 25 | 4 |

Data taken from politigenomics.

The main characteristics of the reads produced by these technologies are:

454
:   454 produces a lower coverage than Illumina, but longer reads. Due to the nature of the sequencing reaction used the main error in the 454 reads are the insertions and deletion on the homopolymer tracks.

Illumina
:   It produces a great coverage at low cost, but with shorter read lengths. The main error found are the substitutions.

SOLiD
:   It produces a coverage and read length similar to Illumina. The main drawback (or advantage) is that due to its ligation based sequencing reaction it does not renders sequence tracks but color tracks. A lot of software is not ready to deal with this kind of color based result.

# Software¶

The NGS sequences would have no value without the software required to analyze them. Due to its volume and to its quality the software created to deal with the sanger sequences cannot deal with them. The field of the NGS analysis software is evolving at a furious pace. The best place to follow this evolution, to ask for advice and to solve doubts it the internet forum seqanswers.

### Table Of Contents

- Introduction
- Usage
- Naming conventions
- Available analyses
- Parallel operation
- Installation
- Cleaning sequence reads
- Mira assembly
- Mapping
- Bam realignment
- Annotation
- Snv filters
- Tutorials
- NGS workshop
  - Next Generation Sequencing
  - Platforms
  - Software
  - File formats
  - Read Cleaning
  - Task 1: cleaning the reads
  - Task 2: read statistics
  - Assembly vs mapping
  - Mapping
  - sam format
  - sam realignment
  - Task 3: read mapping
  - Task 3: Taking a look at a bam file
  - SNP calling
  - SNP filtering
  - VCF format
  - GFF format
  - Task 4: SNP calling
  - Task 5: Looking at the SNPs using IGV
  - Task 5: SNP filtering
  - Command line primer
- Licence
- Indices and tables
- seq\_io
- Architecture

### Search


Enter search terms or a module, class or function name.

index |
next |
previous
  
Show Source

© Copyright 2010, Jose Blanca.
Created using Sphinx 1.0pre.
