# Supplementary material for: ngs_backbone: a pipeline for read cleaning, mapping and SNP calling using Next Generation Sequence
Source: BMC Genomics. 2011 Jun 2;12:285. doi: 10.1186/1471-2164-12-285 (PMC3124440; doi:10.1186/1471-2164-12-285)
Supplement: Additional file 1 — ngs_backbone 1.1.0 software. ngs_backbone 1.1.0. Last version, released on 31-08-2010. [file 1471-2164-12-285-S1.GZ › ngs_backbone-1.1.0/doc/ngs_workshop/sequence_file_formats.html]

File formats — ngs\_backbone v0.1 documentation


# ngs\_backbone v0.1 documentation

index |
next |
previous

# File formats¶

There are a lot of file sequence file formats. The include different information about the sequence and have a very different aspect. The most common file formats in the NGS world are: sff, fastq and fasta.

## sff¶

The SFF (Standard Flowgram Format) files are the 454 equivalent to the ABI chromatogram files. The hold the information about the flowgram, the called sequence and the recommended quality clipping. These are binary files. We can obtain a more usable sequence text file by using the tools provided by Roche with the 454 machine. Alternatively we can use the sff\_extract <http://bioinf.comav.upv.es/sff\_extract/index.html> tool to obtain a fasta file.

## sanger fastq¶

The fastq format was developed to provided a convenient way of storing the sequence and the quality in the same file. These are text files and the look like:

```
@seq_1
GATTTGGGGTTCAAAGCAGTATCGATCAAATAGTAAATCCATTTGTTCAACTCACAGTTT
+
!''*((((***+))%%%++)(%%%%).1***-+*''))**55CCF>>>>>>CCCCCCC65
@seq_2
ATCGTAGTCTAGTCTATGCTAGTGCGATGCTAGTGCTAGTCGTATGCATGGCTATGTGTG
+
208DA8308AD8SF83FH0SD8F08APFIDJFN34JW830UDS8UFDSADPFIJ3N8DAA
```

In this file every sequence has 4 lines. In the first line we get the name and, optionally, the description after the symbol “@”. The second line has the sequence and the fourth line has the quality scores encoded as letters.

## illummina fastq¶

This file is almost identical to a sanger fastq file, but the encoding for the quality scores is different. When we deal with a fastq file we have to be sure about which kind of file we are dealing with, an illumina fastq or a sanger fastq. Unfortunately they are not easy to distinguish. Also you have to take into account that solexa used to had a third fastq format, the solexa fastq.

### Table Of Contents

- Introduction
- Usage
- Naming conventions
- Available analyses
- Parallel operation
- Installation
- Cleaning sequence reads
- Mira assembly
- Mapping
- Bam realignment
- Annotation
- Snv filters
- Tutorials
- NGS workshop
  - Next Generation Sequencing
  - Platforms
  - Software
  - File formats
  - Read Cleaning
  - Task 1: cleaning the reads
  - Task 2: read statistics
  - Assembly vs mapping
  - Mapping
  - sam format
  - sam realignment
  - Task 3: read mapping
  - Task 3: Taking a look at a bam file
  - SNP calling
  - SNP filtering
  - VCF format
  - GFF format
  - Task 4: SNP calling
  - Task 5: Looking at the SNPs using IGV
  - Task 5: SNP filtering
  - Command line primer
- Licence
- Indices and tables
- seq\_io
- Architecture

### Search


Enter search terms or a module, class or function name.

index |
next |
previous
  
Show Source

© Copyright 2010, Jose Blanca.
Created using Sphinx 1.0pre.
