# Supplementary material for: ngs_backbone: a pipeline for read cleaning, mapping and SNP calling using Next Generation Sequence
Source: BMC Genomics. 2011 Jun 2;12:285. doi: 10.1186/1471-2164-12-285 (PMC3124440; doi:10.1186/1471-2164-12-285)
Supplement: Additional file 1 — ngs_backbone 1.1.0 software. ngs_backbone 1.1.0. Last version, released on 31-08-2010. [file 1471-2164-12-285-S1.GZ › ngs_backbone-1.1.0/doc/ngs_workshop/read_cleaning.html]

Read Cleaning — ngs\_backbone v0.1 documentation


# ngs\_backbone v0.1 documentation

index |
next |
previous

# Read Cleaning¶

Before using the raw sequences generated by the sequencing machines it is advisable to clean them to get rid of vector and low quality regions. During the cloning and sequencing processes several vectors and adaptors are usually added to the sequences. If we were to use these raw sequences these vectors could interfere with the rest of the analyses. If we know for sure that the software that we are using is prepared to deal with these vectors we could go on without the cleaning, but in general we do it.

Some people advise against the low quality trimming of the sequences, because even the low quality regions have information in them. It would be advisable to keep these regions if the analysis software would not be affected by them, but in most instances this is not the case.

## Vectors¶

It is not common to find cloning vectors in the NGS sequences because the cloning step is skip in most of these experiments. They are more common in the sanger sequences. If we have them we can look for them and remove them. There are two main approaches to find them. If we know the exact vector and cloning site sequence we could use lucy. lucy looks carefully for the cloning sites and for the given vector and recommend how to trim the sequences. If we are not sure about which sequencing vector was used we could blast our reads against the UniVect database and trim the regions with significant blast matches.

ngs\_backbone uses both approaches, lucy when the vector and cloning site is known and blast with UniVec for the rest.

## Adaptors¶

The main difference between the vectors and adaptors is the sequence length, the adaptors are short sequences. To look for them blast is not the best approach, it is better to do an alignment between them and the reads by using an aligner like exonerate or SSAHA.

When the adaptors are shorter than 15 base pairs the algorithms use by the aligners might fail. An alternative in these case it to look for exact matches.

ngs\_backbone uses exonerate for the adaptors with more than 15 bases and a regular expression search for the shorter ones.

## Quality¶

The usual approach to get rid of the low quality region is to do a window analysis setting a threshold for the quality. ngs\_backbone uses this approach by using lucy with the longer sequences and it own implementation with the shorter ones.

# Task 1: cleaning the reads¶

For this workshop we are going to use the software ngs\_backbone. We are going to clean and map some 454, illumina and sanger sequences and we are going to look for SNPs in them. As a real world example we have prepared some public maize reads obtained from the SRA NCBI section. These new sequences are not in the usual Genbank sections. The NCBI has created a new section called SRA (Sequence Read Archive) prepared for these sequences.

## The raw sequences¶

The sequences downloaded from the SRA are in sanger fastq format. This will not be the case if you get a file from a sequencing service. The 454 machine delivers sff files, illumina illumina fastq files and the ABI sanger sequencers abi chromatograms. Take a look at the sequence *file formats* to understand their differences and how to go from one format to another format. Since we have got our sequences from the SRA site all our reads are in sanger fastq format.

The first step is to download the sequence files. Open a terminal and uncompress the file with the command:

```
$ tar -xzf ngs_workshop.tar.gz
```

Luckily we should have a directory named ngs\_workshop with a subdirectory named course\_data.

```
$ cd ngs_workshop
$ ls course_data/
lb_various.pl_sanger.sm_various.clean_reads.fasta
maize_unigenes.fasta
sm_B73.lb_B73_root.pl_454.raw_reads.sfastq
sm_B73XMo17.lb_MB_14day.pl_illumina.clean_reads.sfastq
```

In this directory we have three files with reads, two of them have been already cleaned (or we just don’t want to clean them) and one is raw, as it came from the sequencing machine. So the first thing to do is to clean these reads. We will do it by using ngs\_backbone.

Also the final result is also available, just in case you want to check anything.

## The cleaning¶

To use ngs\_backbone we have to create a new project, We just run the command:

```
$ backbone_create_project.py -p maize
```

Now we should have new maize directory. In this directory there is a file:

```
$ ls maize/
backbone.conf
```

The backbone.conf file holds the configuration for the different analyses done by ngs\_backbone.

Now we create a directory named reads with a subdirectory named raw and we copy to this directory the file to clean:

```
~/ngs_workshop/maize$ cd maize/
~/ngs_workshop/maize$ mkdir reads
~/ngs_workshop/maize$ mkdir reads/raw
~/ngs_workshop/maize$ cp ../course_data/sm_B73XMo17.lb_MB_14day.pl_illumina.sfastq reads/raw/
~/ngs_workshop/maize$ cp ../course_data/sm_mix.lb_genbank.pl_sanger.fasta reads/raw/
~/ngs_workshop/maize$ ls reads/raw/
sm_B73XMo17.lb_MB_14day.pl_illumina.sfastq  sm_mix.lb_genbank.pl_sanger.fasta
```

Take into account that the read file name is quite special. This is a requirement of ngs\_backbone, in this file name we have the information about the library (lb), sample(sm) and platform (454). ngs\_backbone is quite strict about file and directory names, you can take a look at its file naming conventions in its documentation.

Once we have everything in place we could run the cleaning analysis, but it would be wise though to take a look at the configuration file. There are some default parameters for the cleaning in there, but we might have to adapt them to our needs. You can take a look at this parameters in the configuration file by using a text editor and to better understand their meaning to go the cleaning section of the backbone documentation. Before runing the analysis at least make sure that the vector\_database points to the Univec blast database. Once everything is set up just run the analysis:

```
(devel)~/ngs_workshop/maize$ backbone_analysis.py -a clean_reads
2010-05-25 15:19:38,183 INFO CleanReadsAnalyzer
2010-05-25 15:19:38,184 INFO backbone version: 0.1.0
2010-05-25 15:19:38,184 INFO Analysis started
2010-05-25 15:20:06,123 INFO Analysis finished
INFO:franklin:Analysis finished
2010-05-25 15:20:06,124 INFO Time elapsed 0:00:27.991122
INFO:franklin:Time elapsed 0:00:27.991122
```

ngs\_backbone loads the configuration file and does the analysis using those parameters. Once the analysis is done you will find several new files in the project directory:

backbone.log
:   Everything done by ngs\_backbone will be logged in this file. If we remove the file the program will create a new one the next time is run.

tmp
:   The temp directory in which all the temporary file are stored. Once the analysis is done you can safely remove this directory. You can also set which directory to use as temp in backbone.conf.

Also a new directory named reads/cleaned will be created with the new cleaned files in it.

You can take a look at the files with any text editor. For instance you can find something like:

```
>FM195262.1 FM195262 ZEASTAR-B Zea mays cDNA clone ZEASTAR-B-003-C03, mRNA sequence.
TACGGCCGGGGTNNCNNANNNNGCATTCTCGCAGGGTCTTTCTACACTATTAGATAAGAT
GGATCCTTCTCAGAGAGTGAAGTTTGTTCAGGAAGTCAAGAAGGTTCTTGGATGATGATA
TGATACCAACACATCCAACACAATATGCGCATGCTACATGTTATTTTTCAAGTACATACA
TAGAAGGATATTGCTTGGCCTTGATTGATCATGTCTGATCTAAGTCGATCATTATTTTCT
TGAAACTTCCTTTCGGACGTGGTGCTATGGTTGATGAATTTGGATGTGTGCGTTCTGCCA
GGTGTAAGCCCAAAGGTTTATACAGACCGAGTTAAGGTTAGGAAGAGCACGAGTGAACTT
GTTCTGGTTTTGCAGTGGTTAAGGCAGAAAGTTGTTTCACTGTAGTTCTGAGATGTATTA
CCAGCGGCGCTGTAATTTTAGGGTGTATAATGCGGATGCTAGTAAACAATTGAGTGGTTC
ATTAAATTTTGAACTCGAATAATATGTTTCTGTAGATA
```

As you can see not all the bad quality regions has been removed. If you want a more stringent cleaning for this reads you can change the relevant parameters in the backbone.conf file, delete the cleaned file and rerun the analysis.

Open the backbone.conf with a text editor and, in the cleaning section, change the value strip\_n\_percent. You can try with 0.5 or 5.0 and take a look at the result. Once that you have edited the backbone.conf remove the old cleaned file and do the analysis again.

```
~/ngs_workshop/maize$ rm reads/cleaned/sm_mix.lb_genbank.pl_sanger.fasta
~/ngs_workshop/maize$ backbone_analysis.py -a clean_reads
2010-05-26 07:51:10,327 INFO CleanReadsAnalyzer
2010-05-26 07:51:10,328 INFO backbone version: 0.1.0
2010-05-26 07:51:10,328 INFO Analysis started
2010-05-26 07:51:10,329 INFO /home/jose/ngs_workshop/maize/reads/cleaned/sm_B73XMo17.lb_MB_14day.pl_illumina.sfastq already cleaned. Not cleaned again
2010-05-26 07:51:36,410 INFO Analysis finished
INFO:franklin:Analysis finished
2010-05-26 07:51:36,411 INFO Time elapsed 0:00:26.128035
INFO:franklin:Time elapsed 0:00:26.128035
```

This time ngs\_backbone warn us that the illumina sequence are not cleaned again because a cleaned file was already found there. Every time this analysis is run only the files not yet cleaned are cleaned. For instance, we removed the sanger fasta file and redoing the analysis does clean this file again. With strip\_n\_percent set to 0.5 the result sequences now look like:

```
>FM195262.1 FM195262 ZEASTAR-B Zea mays cDNA clone ZEASTAR-B-003-C03, mRNA sequence.
GCATTCTCGCAGGGTCTTTCTACACTATTAGATAAGATGGATCCTTCTCAGAGAGTGAAG
TTTGTTCAGGAAGTCAAGAAGGTTCTTGGATGATGATATGATACCAACACATCCAACACA
ATATGCGCATGCTACATGTTATTTTTCAAGTACATACATAGAAGGATATTGCTTGGCCTT
GATTGATCATGTCTGATCTAAGTCGATCATTATTTTCTTGAAACTTCCTTTCGGACGTGG
TGCTATGGTTGATGAATTTGGATGTGTGCGTTCTGCCAGGTGTAAGCCCAAAGGTTTATA
CAGACCGAGTTAAGGTTAGGAAGAGCACGAGTGAACTTGTTCTGGTTTTGCAGTGGTTAA
GGCAGAAAGTTGTTTCACTGTAGTTCTGAGATGTATTACCAGCGGCGCTGTAATTTTAGG
GTGTATAATGCGGATGCTAGTAAACAATTGAGTGGTTCATTAAATTTTGAACTCGAATAA
TATGTTTCTGTAGATA
```

# Task 2: read statistics¶

Once we have cleaned the sequences it would be nice to get statistics about the raw and the cleaned sequences. We can do it running the following analysis:

```
~/ngs_workshop/maize$ backbone_analysis.py -a read_stats
2010-05-21 09:49:20,462 INFO ReadsStatsAnalyzer
2010-05-21 09:49:20,462 INFO backbone version: 0.1.0
2010-05-21 09:49:20,462 INFO Analysis started
2010-05-21 09:50:36,396 INFO Analysis finished
2010-05-21 09:50:36,481 INFO Time elapsed 0:01:16.060827
```

Once the analysis is done we will have a stats subdirectory in the reads/raw and in the reads/cleaned directory. If we go to one of these directories we will find several text files and charts. The results are:

seq\_length\_distrib
:   Sequence length distribution of the reads.

qual\_distrib
:   Phred quality values distribution.

general\_stats
:   Some general statistics like, total length, length and quality range and variance.

These statistics are generated for every read file found in the raw and cleaned directories.

For each chart ngs\_backbone creates two files, one with the figure and another with the data used to build the chart.

If for a given file both the cleaned and the raw versions are found a difference distribution for the cleaned and the raw statistics are also generated.

### Table Of Contents

- Introduction
- Usage
- Naming conventions
- Available analyses
- Parallel operation
- Installation
- Cleaning sequence reads
- Mira assembly
- Mapping
- Bam realignment
- Annotation
- Snv filters
- Tutorials
- NGS workshop
  - Next Generation Sequencing
  - Platforms
  - Software
  - File formats
  - Read Cleaning
  - Task 1: cleaning the reads
  - Task 2: read statistics
  - Assembly vs mapping
  - Mapping
  - sam format
  - sam realignment
  - Task 3: read mapping
  - Task 3: Taking a look at a bam file
  - SNP calling
  - SNP filtering
  - VCF format
  - GFF format
  - Task 4: SNP calling
  - Task 5: Looking at the SNPs using IGV
  - Task 5: SNP filtering
  - Command line primer
- Licence
- Indices and tables
- seq\_io
- Architecture

### Search


Enter search terms or a module, class or function name.

index |
next |
previous
  
Show Source

© Copyright 2010, Jose Blanca.
Created using Sphinx 1.0pre.
