# Supplementary material for: ngs_backbone: a pipeline for read cleaning, mapping and SNP calling using Next Generation Sequence
Source: BMC Genomics. 2011 Jun 2;12:285. doi: 10.1186/1471-2164-12-285 (PMC3124440; doi:10.1186/1471-2164-12-285)
Supplement: Additional file 1 — ngs_backbone 1.1.0 software. ngs_backbone 1.1.0. Last version, released on 31-08-2010. [file 1471-2164-12-285-S1.GZ › ngs_backbone-1.1.0/doc/ngs_workshop/snp_calling.html]

SNP calling — ngs\_backbone v0.1 documentation


# ngs\_backbone v0.1 documentation

index |
next |
previous

# SNP calling¶

One of the main applications of the NGS technologies is the SNP mining in the resequencing projects. Sequences from different individuals are generated and we look for Single Nucleotide Polymorphisms comparing them with the reference genome.

Once we have an alignment as a bam file looking for SNPs is not a conceptually difficult task. We go through every column of the alignment and in every one we see how many alleles we found and how they compare with the one found in the reference genome. This naive view is complicated by several factors:

> - The error rate associated with the reads.
> - The error rate associated with the mapping.
> - The reliability of the reference genome.

Every base on the reads is generated with a phred score associated. This score relates to the error probability of the base. A base with a low score might be just a sequencing error and not a real variation.

Also, the mapping tools calculate a probability for the correctness of the alignment. This probability depends on the length of alignment, on the number of mismatches and gaps and on the uniqueness of the aligned region on the genome. It is important to distinguish the real SNPs from the mismatches between repeated homologous genomic regions.

Once that we are sure that our allele is not a sequencing error and that we have not misalign two genomic repeats we can face another hurdle. Depending on the quality of the reference genome if we found an allele different we can decide that it is a real variation or that might just be a sequencing error in the reference genome.

Once we have a sam file we can use several tools that call the SNPs (e.g. PolyBayes, GATK or ngs\_backbone). Each one of these SNP callers make different assumptions about the reference genome and the reads, so each one of them is best suited for different situations.

# SNP filtering¶

Usually we do not require as many as SNPs as we get from the NGS sequences. One way to reduce the number of SNPs is to choose some of them at random. This simple approach would be good if all SNPs had the same usefulness, but this is not the case. Some SNPs are more polymorphic than other, or are more easily detected by PCR or by a restriction enzyme or can be located in a gene of interest. There are a lot of different criteria to segregate the SNPs, ngs\_backbone includes some of these filters.

# VCF format¶

The end result of a SNP calling analysis is a collection of SNPs, and an standard file have been created to hold this SNPs, the Variant Call Format file (VCF). In this file every line represents an SNP and the following information is found:

> - The position in the reference genome
> - The allele in the reference genome
> - The other alleles found
> - The filters not passed by the SNP
> - The genotypes found with its abundances

There are not yet too many tools capable of dealing with these files, but they are text files and we can take a look at them quite easily with a text editor. Also, the fields in this file are delimited by tabs, so it can be imported into a spreadsheet program using the csv option.

# GFF format¶

The GFF files are used to store annotations. The SNPs are one of such annotations, but there are much more (introns, ORFs, UTRs, etc.). The GFF files are text files and every line represents a region on the annotated sequence and these regions are called features. Each of these features have a kind associated. The terms used to define these kinds should belong to the Sequence Ontology terms. If you are interested you can take a look at the Sequence Ontology or at the GFF format specification.

# Task 4: SNP calling¶

Calling the SNPs with ngs\_backbone is very easy, but before doing it take a look at the Snvs section in the backbone.conf file:

```
[Snvs]
  min_quality = 45
  min_mapq = 15
  min_num_alleles = 2
  [[edge_removal]]
      454_left = None
      454_right = None
      sanger_left = None
      sanger_right = None
      illumina_left = None
      illumina_right = None
```

The parameters are:

min\_quality
:   The quality threshold used to define what is considered an allele. This parameter is used to differentiate the sequencing errors from the real alleles.

min\_mapq
:   When we call the SNPs not all mapped reads are used, only the ones with a mapping quality above this minimum are used. The others could be due, for instance, to a duplicated region and we don’t want to treat differences in the duplicated regions as SNPs.

min\_num\_alleles
:   There are two main criteria when defining a SNP. A SNPs is any position in which we find two different alleles or an SNPs is any position with an allele different to the one found in the reference genome. To opt for the first option we should do min\_num\_alleles equal to two.

edge\_removal
:   If we don’t trust the read edges we could ignore several base pairs by using these parameters.

We want only SNPs with at least two alleles so, edit the configuration file and, in the Snvs section, we set the min\_num\_alleles to 2.

ngs\_backbone considers the SNP calling as a form of annotation. What we are doing is annotating the maize transcriptome with the SNPs. To do an annotation with ngs\_backbone we have to create an annotation directory and to put in the input subdirectory the files to annotate, in this case some maize unigenes.

```
~/ngs_workshop/maize$ mkdir annotations
~/ngs_workshop/maize$ mkdir annotations/input
~/ngs_workshop/maize$ cp ~/ngs_workshop/course_data/maize_unigenes.fasta annotations/input/
~/ngs_workshop/maize$ ls annotations/input/
maize_unigenes.fasta
```

Now that we have the file to annotate and the bam file in place we can call the SNPs.

```
~/ngs_workshop/maize$ backbone_analysis.py -a annotate_snvs
2010-05-26 08:09:17,599 INFO SnvCallerAnalyzer
2010-05-26 08:09:17,599 INFO backbone version: 0.1.0
2010-05-26 08:09:17,599 INFO Analysis started
2010-05-26 08:11:09,948 INFO Analysis finished
INFO:franklin:Analysis finished
2010-05-26 08:11:09,949 INFO Time elapsed 0:01:52.395800
INFO:franklin:Time elapsed 0:01:52.395800
```

Once the analysis is finished we will have a file in the directory annotations/db/. These files hold all the annotations done. We could now annotate the ORFs and a new db file would be generated with that annotation added. These files are versioned, it is safe to removed all of them but the last one.

To get the real annotations result files we have to do one last analysis:

```
~/ngs_workshop/maize$ backbone_analysis.py -a write_annotations
2010-05-21 12:03:29,074 INFO Time elapsed 0:00:04.748977
```

This analysis will generate several output files in the directory annotation/features/. In this case we get a GFF file and a VCF file.

```
~/ngs_workshop/maize$ ls annotations/features/
maize_unigenes.gff3  maize_unigenes.vcf
```

We will use the GFF file to take a look at the called SNPs in IGV. In the VCF file we can find the information regarding the SNPs. Open it and take a look at it, remember to take a look also at the VCF specification. In this file we have the information about:

> - where is the SNP.
> - which are the alleles.
> - how many times each allele has been found in every read group.
> - which filters has the SNP fail to pass.

# Task 5: Looking at the SNPs using IGV¶

In the IGV we can load the bam and the GFF. In that way we can compare the mapping with the annotation. To do it, open IGV and load the bam file. This time you won’t need to import the reference transcriptome, it will be automatically selected, because it was the last reference used. Load the bam file as you did the last time. Also load the gff file in annotation/features/. Now you should have two tracks in IGV, one with the mapping and another one with the annotation. In this case we have just annotated the SNPs, but ORFs, SSR or whatever other annotation would also appear here.

Sometimes IGV shows the GFF track collapsed by default, you can expand it by clicking on the right mouse button above the track and selecting expand.

# Task 5: SNP filtering¶

Now that we have some SNPs we can select which ones are the best for us. In ngs\_backbone we can filter SNPs according to several criteria. Here we will just use a couple of them. Let’s filter the SNPs that are really SNPs and are not indels and the ones that are in a highly variable region (that might be due to misalignments). To do it we have to edit the snv\_filters section in the backbone.conf file. There you will find several examples filters configured as an example. None of these filters are used because they all have the parameter use set to False. To set up our two filters we have to write in the configuration file:

```
[[filter3]]
    name = 'high_variable_region'
    use = True
    max_variability = 0.06
    window = None
[[filter7]]
    name = 'by_kind'
    use = True
    kind = 'SNP'
```

Now we run the analysis:

```
~/ngs_workshop/maize$ backbone_analysis.py -a filter_snvs
2010-05-26 15:55:41,259 INFO SnvFilterAnalyzer
2010-05-26 15:55:41,259 INFO backbone version: 0.1.0
2010-05-26 15:55:41,259 INFO Analysis started
2010-05-26 15:55:42,702 INFO Analysis finished
INFO:franklin:Analysis finished
2010-05-26 15:55:42,702 INFO Time elapsed 0:00:01.520483
INFO:franklin:Time elapsed 0:00:01.520483
```

And we write the VCF file again:

```
~/ngs_workshop/maize$ backbone_analysis.py -a write_annotations
2010-05-26 15:56:18,004 INFO Time elapsed 0:00:01.475464
```

Now the VCF will have the filter column filled:

```
##FILTER=HVR6,"The region has more than 6 snvs per 100 bases"
##FILTER=VKS,"It filters if it is of kind: snp"
#CHROM  POS     ID      REF     ALT     QUAL    FILTER
TC458159        1491    .       A       D1,G,T  138     HVR6;VKS
TC458158        7104    .       G       T       55      .
TC458159        29      .       C       T       50      HVR6
```

The meaning of the tags that appear in the filter column is explained at the beginning of the file. When one tag appears in the filter column (like VKS) means that this SNP has not passed that filter, in this case it is not an SNP but an indel. An SNP that passed all filters should have a “.” in the filter column. In the example shown the SNP in the position 7104 passes all filters, it is an SNP and it is not in a highly variable region.

### Table Of Contents

- Introduction
- Usage
- Naming conventions
- Available analyses
- Parallel operation
- Installation
- Cleaning sequence reads
- Mira assembly
- Mapping
- Bam realignment
- Annotation
- Snv filters
- Tutorials
- NGS workshop
  - Next Generation Sequencing
  - Platforms
  - Software
  - File formats
  - Read Cleaning
  - Task 1: cleaning the reads
  - Task 2: read statistics
  - Assembly vs mapping
  - Mapping
  - sam format
  - sam realignment
  - Task 3: read mapping
  - Task 3: Taking a look at a bam file
  - SNP calling
  - SNP filtering
  - VCF format
  - GFF format
  - Task 4: SNP calling
  - Task 5: Looking at the SNPs using IGV
  - Task 5: SNP filtering
  - Command line primer
- Licence
- Indices and tables
- seq\_io
- Architecture

### Search


Enter search terms or a module, class or function name.

index |
next |
previous
  
Show Source

© Copyright 2010, Jose Blanca.
Created using Sphinx 1.0pre.
