# Supplementary material for: ngs_backbone: a pipeline for read cleaning, mapping and SNP calling using Next Generation Sequence
Source: BMC Genomics. 2011 Jun 2;12:285. doi: 10.1186/1471-2164-12-285 (PMC3124440; doi:10.1186/1471-2164-12-285)
Supplement: Additional file 1 — ngs_backbone 1.1.0 software. ngs_backbone 1.1.0. Last version, released on 31-08-2010. [file 1471-2164-12-285-S1.GZ › ngs_backbone-1.1.0/doc/ngs_workshop/assembly_vs_mapping.html]

Assembly versus mapping — ngs\_backbone v0.1 documentation


# ngs\_backbone v0.1 documentation

index |
next |
previous

# Assembly versus mapping¶

Once we have a collection of sequence reads there are two different kinds of analysis. If we do not have any previous genomic information we would have to assemble the reads into a genome or transcriptome. Alternatively, if we had genome already available we could map our reads against that genome. Although both analyses could seem to be similar they are very different. To assemble a genome is computationally much costly than to do a mapping, assembling the human genome was a difficult task, re-sequencing and mapping the reads from a new individual is a more amenable task.

The main computational difference is that the typical software used to assemble requires a time that depends on the total reads length squared while the mapping is just lineal with the reads length. For a review take a look at Sense from sequence reads, but the take home message is that the assembly is time and memory consuming while the mapping can be done in standard computer.

Also is it important to notice that the read length is a critical parameter for the assemblies. To assemble a genome with repetitive DNA we need reads longer than the longest repeat, otherwise the assemble would not be able to correctly assembly the repeats. Thus, the shorter the reads the more challenging to do the assembly. If we want to assemble a new genome or transcriptome we would have a much easier time if we are dealing with sanger or 454 reads.

### Table Of Contents

- Introduction
- Usage
- Naming conventions
- Available analyses
- Parallel operation
- Installation
- Cleaning sequence reads
- Mira assembly
- Mapping
- Bam realignment
- Annotation
- Snv filters
- Tutorials
- NGS workshop
  - Next Generation Sequencing
  - Platforms
  - Software
  - File formats
  - Read Cleaning
  - Task 1: cleaning the reads
  - Task 2: read statistics
  - Assembly vs mapping
  - Mapping
  - sam format
  - sam realignment
  - Task 3: read mapping
  - Task 3: Taking a look at a bam file
  - SNP calling
  - SNP filtering
  - VCF format
  - GFF format
  - Task 4: SNP calling
  - Task 5: Looking at the SNPs using IGV
  - Task 5: SNP filtering
  - Command line primer
- Licence
- Indices and tables
- seq\_io
- Architecture

### Search


Enter search terms or a module, class or function name.

index |
next |
previous
  
Show Source

© Copyright 2010, Jose Blanca.
Created using Sphinx 1.0pre.
