# Supplementary material for: ngs_backbone: a pipeline for read cleaning, mapping and SNP calling using Next Generation Sequence
Source: BMC Genomics. 2011 Jun 2;12:285. doi: 10.1186/1471-2164-12-285 (PMC3124440; doi:10.1186/1471-2164-12-285)
Supplement: Additional file 1 — ngs_backbone 1.1.0 software. ngs_backbone 1.1.0. Last version, released on 31-08-2010. [file 1471-2164-12-285-S1.GZ › ngs_backbone-1.1.0/doc/architecture.html]

Architecture — ngs\_backbone v0.1 documentation


# ngs\_backbone v0.1 documentation

index |
previous

# Architecture¶

This section is intended to be read by python developers interested in fixing bugs or extending the functionality of ngs\_backbone. A standard user does not need to read it.

The ngs\_backbone functionality depends heavily on Biopython and on some external tools, these are the foundations that ngs\_backbone requires to do its work. The functions and classes provided by franklin itself are collected in two main packages: franklin and backbone.

The package franklin provides the required functions to do the different analysis. The backbone package (located in franklin/backbone) is used to build the user interface to the application. For instance in the cleaning analysis:
:   - the backbone will look at the configuration file, it will look for the input and output paths,
    - and the franklin package has the functions that read the files, clean the sequence objects (using several external applications), and write the output sequences.

## franklin package architecture¶

In franklin all the analyses are done on sequences so the main class is SeqWithQuality (franklin/seq/seqs.py). SeqWithQuality is just a Biopython SeqRecord with some modifications. These SeqWithQuality sequences when a sequence file is read by the function seqs\_in\_file (franklin/seq/readers.py). Once the analysis is completed the resulting sequence is written to the output by a writer class named SequenceWriter (franklin/seq/writer.py).

An analysis is represented by a pipeline composed by several steps. All the analyses are run by the same function: seq\_pipeline\_runner (franklin/pipelines/pipelines.py). This function creates a list of analysis steps and a configuration for them and runs them. Each step is composed by a function and a configuration. These analysis step functions are classified in three types: mappers, filters and bulk mappers. The mapper and filter take a SeqWithQuality as input and generate either a new SeqWithQuality (the mapper) or a boolean (the filter). The bulk mappers take a stream of SeqWithQuality sequences and return a new streams.

The analysis steps are entries in a python dictionary that define the analysis function, the kind of function and the description of the analysis. These analysis steps are defined in several modules in the fraklin/pipelines package like seq\_pipeline\_steps.py (sequence cleaning), annotation\_steps.py (sequence annotation) and snv\_pipeline\_steps.py (snv calling and filtering).

franklin is designed to deal with millions of sequences. Since most of the analysis performed can be done for each sequence independently only once sequence is keep in memory at each time. This is accomplished by using python generators, the sequence streams are in fact a python generator that yield one SeqWithQuality at a time. The function seqs\_in\_file is the main of such generators.

### Table Of Contents

- Introduction
- Usage
- Naming conventions
- Available analyses
- Parallel operation
- Installation
- Cleaning sequence reads
- Mira assembly
- Mapping
- Bam realignment
- Annotation
- Snv filters
- Tutorials
- NGS workshop
- Licence
- Indices and tables
- seq\_io
- Architecture
  - franklin package architecture

### Search


Enter search terms or a module, class or function name.

index |
previous
  
Show Source

© Copyright 2010, Jose Blanca.
Created using Sphinx 1.0pre.
