# Supplementary material for: ngs_backbone: a pipeline for read cleaning, mapping and SNP calling using Next Generation Sequence
Source: BMC Genomics. 2011 Jun 2;12:285. doi: 10.1186/1471-2164-12-285 (PMC3124440; doi:10.1186/1471-2164-12-285)
Supplement: Additional file 1 — ngs_backbone 1.1.0 software. ngs_backbone 1.1.0. Last version, released on 31-08-2010. [file 1471-2164-12-285-S1.GZ › ngs_backbone-1.1.0/doc/tutorials.html]

Tutorials — ngs\_backbone v0.1 documentation


# ngs\_backbone v0.1 documentation

index |
next |
previous

# Tutorials¶

In the tutorials we will do several analyses step by step. To understand how those analyses are done it would be advisable to read the documents *Cleaning sequence reads* and *Mapping* before or after going through the tutorials. This tutorial is a brief introduction, for a more lengthly document you can take a look at the ngs\_workshop *notes*.

## Cleaning sequences¶

We have some sanger, 454 and illumina reads and we want to clean them and to do some quality control after the process is finished.

The first step is to create a ngs\_backbone project.

```
$ backbone_create_project.py -p tutorial
$ cd tutorial/
$ head -n 5 backbone.conf
[General_settings]
tmpdir = '/home/jose/tmp/tutorial/tmp'
project_name = 'tutorial'
project_path = '/home/jose/tmp/tutorial'
```

The create\_project scripts makes a directory for the project and creates a configuration file template in it. This file will hold all the information about the parameters required to run the analysis.

For the tutorial I will clean the demo reads distributed with ngs\_backbone, you can find them in franklin/franklin/data/acceptance/. They have to be located at reads/raw/ and they should respect the *naming conventions*. Also take into account that ngs\_backbone only will accept fasta and fastq files.

```
$ mkdir reads
$ mkdir reads/raw
$ cp ~/ngs_backbone-1.0.0/franklin/data/acceptance/cleaning/* reads/raw/
$ ls reads/raw/
lb_microtom_gb.pl_sanger.sm_microtom.fasta
lb_mu16.pl_454.sm_mu16.sfastq
lb_microtom_sgn.pl_sanger.sm_microtom.sfastq
lb_ximo.pl_illumina.sm_rp_75_59_uc82.sfastq
```

Before running the analysis take into account that you will need some external software do it, so be sure that it is installed. The software required for the cleaning is lucy, exonerate, blast, Univec (database), mdust, and trimpoly.

The analysis could be run with the default parameters, but for some cases it would be convenient to tweak the configuration file. For instance it could be of some help to specify which adapters or cloning vectors have been used to build the libraries. To add them take a look at the configuration parameters related to the cleaning *cleaning*. One you have the configuration set up you can run the analysis doing just:

```
$ backbone_analysis.py -a clean_reads
2010-04-28 08:25:03,508 INFO CleanReadsAnalyzer
2010-04-28 08:25:03,509 INFO Franklin VERSION: 0.01
2010-04-28 08:25:03,509 INFO Analysis started
2010-04-28 08:26:14,092 INFO Analysis finished
INFO:franklin:Analysis finished
2010-04-28 08:26:14,093 INFO Time elapsed 0:01:10.697465
INFO:franklin:Time elapsed 0:01:10.697465
$ ls
franklin.conf  franklin.log  reads  tmp
```

Several files and directories have been generated. ngs\_backbone.log is just a log file in which every analysis done will be stored. tmp is a temporal directory that can be safely removed (by the way the location of this temporal directory can be changed in the configuration file. The cleaned sequence files will be located at reads/cleaned/:

```
$ ls reads/cleaned/
lb_microtom_gb.pl_sanger.sm_microtom.fasta
lb_microtom_sgn.pl_sanger.sm_microtom.sfastq
lb_mu16.pl_454.sm_mu16.sfastq
lb_ximo.pl_illumina.sm_rp_75_59_uc82.sfastq
```

To finish up the analysis we can create some statistics about both the raw and the cleaned files doing a new analysis:

```
$ backbone_analysis.py -a read_stats
2010-04-28 08:31:48,404 INFO ReadsStatsAnalyzer
2010-04-28 08:31:48,404 INFO Franklin VERSION: 0.01
2010-04-28 08:31:48,404 INFO Analysis started
2010-04-28 08:32:52,051 INFO Analysis finished
2010-04-28 08:32:52,078 INFO Time elapsed 0:01:03.706581
```

In this case the results will be found in two subdirectories named stats found in reads/raw and reads/clean.

## Looking for SNPs¶

A common ngs\_backbone application is to map some reads against a reference genome and to look for SNPs in them.

Again the first step is to create a ngs\_backbone project.

```
$ backbone_create_project.py -p tutorial
$ cd tutorial/
$ head -n 5 backbone.conf
[General_settings]
tmpdir = '/home/jose/tmp/tutorial/tmp'
project_name = 'tutorial'
project_path = '/home/jose/tmp/tutorial'
```

The inputs required are the cleaned reads and a reference genome. The reads should be located at reads/cleaned and the reference genome at mapping/reference/reference.fasta. For the tutorial we will use the example files distributed with ngs\_backbone.

```
$ mkdir reads
$ mkdir reads/cleaned
$ cp ~/ngs_backbone-1.0.0/franklin/data/acceptance/assembling/lb* reads/cleaned/
$ ls reads/cleaned/
lb_microtom_gb.pl_sanger.sm_microtom.fasta
lb_mu16.pl_454.sm_mu16.sfastq
lb_microtom_sgn.pl_sanger.sm_microtom.sfastq
$ mkdir mapping
$ mkdir mapping/reference
$ cp ~/ngs_backbone-1.0.0/franklin/data/acceptance/mapping/reference.fasta mapping/reference/
$ ls mapping/reference/
reference.fasta
```

Be aware that the reads file naming is important, the library and the sequencing technology will be needed during the analysis and this information will be taken from those names. Go to the *naming conventions* section to read more about it.

Now that we have the inputs set up we can do the mapping using bwa.

```
$ backbone_analysis.py -a mapping
2010-04-28 09:46:16,015 INFO MappingAnalyzer
2010-04-28 09:46:16,016 INFO Franklin VERSION: 0.01
2010-04-28 09:46:16,016 INFO Analysis started
2010-04-28 09:46:22,823 INFO Analysis finished
2010-04-28 09:46:22,824 INFO Time elapsed 0:00:06.844860
$ ls mapping/
20100428_0746/ reference/
$ ls mapping/20100428_0746/
result
$ ls mapping/20100428_0746/bams/by_readgroup/
lb_microtom_gb.pl_sanger.sm_microtom.bam
lb_mu16.pl_454.sm_mu16.bam
lb_microtom_sgn.pl_sanger.sm_microtom.bam
```

The result of the analysis is a timestamped directory with the bam files in mapping/bams/by\_readgroup. The directory is timestamped to allow for different mappings done with different parameters or mapping tools.

For every input read file a bam file has been generated in the directory mapping/bams/by\_readgroup. To continue the analysis we are going to merge all bam files into one merged bam file.

```
$ backbone_analysis.py -a merge_bams
2010-04-28 12:37:52,817 INFO MergeBamAnalyzer
2010-04-28 12:37:52,817 INFO Franklin VERSION: 0.01
2010-04-28 12:37:52,817 INFO Analysis started
2010-04-28 12:37:55,497 INFO Analysis finished
2010-04-28 12:37:55,498 INFO Time elapsed 0:00:02.713902
$ ls mapping/bams
by_readgroup  merged.0.bam
```

The resulting merged bam have all the information from the individual bam. Every bam is now a readgroup inside the merged bam. Every readgroup holds the information about its sample, platform and library. In this step the resulting bam file has also been sorted and made picard compatible.

The next step is to realign the bam file using GATK. This step is optional and can be skipped. You have more information about this analysis in the GATK site.

```
$ backbone_analysis.py -a realign_bam
2010-04-28 13:00:38,147 INFO RealignBamAnalyzer
2010-04-28 13:00:38,148 INFO Franklin VERSION: 0.1.0
2010-04-28 13:00:38,148 INFO Analysis started
2010-04-28 13:01:42,884 INFO Analysis finished
2010-04-28 13:01:42,884 INFO Time elapsed 0:01:04.775819
$ ls mapping/bams/
by_readgroup/
merged.0.bam
merged.0.bam.bai
merged.1.bam
```

The result is the file merged.1.bam. The file merged.bam is versioned in the ngs\_backbone system. Several versions of the same file can be located on the same directory and only the last one will be used for the following analysis. The old files can be safely deleted.

Now we want to annotate some sequences with the SNPs found when comparing the mapped reads from the bam file. To do that we have to put the sequences to annotate in annotations/input. Let’s annotate the reference sequence with the SNPs.

```
$ mkdir annotations
$ mkdir annotations/input
$ cd annotations/input
$ ln -s ../../mapping/reference/reference.fasta .
$ backbone_analysis.py -a annotate_snvs
2010-04-28 14:36:18,593 INFO SnvCallerAnalyzer
2010-04-28 14:36:18,593 INFO Franklin VERSION: 0.1.0
2010-04-28 14:36:18,594 INFO Analysis started
2010-04-28 14:37:17,509 INFO Analysis finished
INFO:franklin:Analysis finished
2010-04-28 14:37:17,509 INFO Time elapsed 0:00:58.963646
INFO:franklin:Time elapsed 0:00:58.963646
$ ls annotations/db/
reference.0.pickle
```

The result is the file found in the annotations/db/ directory. This is again a versioned file that holds all the information about the annotated sequences. This file is of no direct interest because its format is quite cumbersome. Every time that we do an annotation analysis a new version of this file will be generated. To get the real result files after doing all the annotations required we do a final analysis.

```
~/personal/devel/franklin/scripts/backbone/backbone_analysis.py -a write_annotation
2010-04-28 14:42:42,361 INFO Time elapsed 0:00:00.289912
$ ls annotations/features/
reference.gff  reference.vcf
```

Now we the results files are found in annotations/features/. In this case a GFF and a vcf files have been generated.

### Table Of Contents

- Introduction
- Usage
- Naming conventions
- Available analyses
- Parallel operation
- Installation
- Cleaning sequence reads
- Mira assembly
- Mapping
- Bam realignment
- Annotation
- Snv filters
- Tutorials
  - Cleaning sequences
  - Looking for SNPs
- NGS workshop
- Licence
- Indices and tables
- seq\_io
- Architecture

### Search


Enter search terms or a module, class or function name.

index |
next |
previous
  
Show Source

© Copyright 2010, Jose Blanca.
Created using Sphinx 1.0pre.
