# Supplementary material for: ngs_backbone: a pipeline for read cleaning, mapping and SNP calling using Next Generation Sequence
Source: BMC Genomics. 2011 Jun 2;12:285. doi: 10.1186/1471-2164-12-285 (PMC3124440; doi:10.1186/1471-2164-12-285)
Supplement: Additional file 1 — ngs_backbone 1.1.0 software. ngs_backbone 1.1.0. Last version, released on 31-08-2010. [file 1471-2164-12-285-S1.GZ › ngs_backbone-1.1.0/doc/introduction.html]

Introduction — ngs\_backbone v0.1 documentation


# ngs\_backbone v0.1 documentation

index |
next |
previous

# Introduction¶

ngs\_backbone is an easy to use application capable of doing in a reliable way some NGS analyses. The main design directives have been:

> - analyses reproducibility
> - ease of use
> - modularity
> - standard format output

By using ngs\_backbone we can run a complete analysis in a reproducible way. Every analysis parameter is configured in a text file and a log file is generated as the analysis progresses. Also the analysis is easy to do, instead of running multiple scripts and programs to do an analysis only one command is required.

An application like that in a fast moving field, as the NGS is, has the risk of stagnate rapidly. To avoid this pitfall special care has been taken in the design of the architecture of ngs\_backbone. Everything within the application is modular and several independent layers have been differentiated within the code to facilitate the maintenance of the code. In fact before its public release ngs\_backbone has gone through refactorings of several modules that have not affected the overall structure of the application.

# Usage¶

The main ideas to consider when using ngs\_backbone are: project and analysis. A *project* is a directory (with its subdirectories) that includes a configuration file and all input and output files. An *analysis* takes some inputs from the project and creates some outputs. ngs\_backbone knows where the input and output files are for every analysis because the project directory structure is the same for every project. For instance the reads to clean are always in the directory /reads/original and the cleaned reads always are in /reads/cleaned/. The main directories in this standard directory layout are:

> - reads/raw
> - reads/cleaned
> - assembly/result
> - annotations/input
> - annotations/blast
> - annotations/features
> - mapping/reference
> - mapping/bams
> - tmp/

The configuration parameters required for every analysis are stored in the configuration file (ngs\_backbone.conf) located in the project directory. A sample of this configuration file is created by ngs\_backbone when a new project is created. This file should be tweaked to adapt the analyses to your requirements before running them.

ngs\_backbone has only two main executables: backbone\_create\_project.py and backbone\_analysis.py. The first one is used to create a project from scratch and the second one to run all analyses.

## Creating a new project¶

```
$ backbone_create_project.py -p project_name
$ ls -l project_name
total 4
-rw-r--r-- 1 jose jose 2479 abr 16 09:30 ngs_backbone.conf
```

This command will create a new directory named project\_name with a file named ngs\_backbone.conf in it. These are the two hallmarks that define a ngs\_backbone *project*, the directory and the configuration file.

## Running an analysis¶

```
$ backbone_analysis.py -a analysis_name
```

This command will run the analysis on the data present in the project using the parameters found in the configuration file. The output files will also be located in the project.

# Naming conventions¶

The ngs\_backbone usage is heavily based on directory and file name conventions. If the input files are not located where ngs\_backbone expects to find them or they have non-standard file names the analysis will fail.

| file or directory type | location |
| --- | --- |
| configuration file | ngs\_backbone.conf |
| log file | ngs\_backbone.log |
| raw reads | reads/raw/ |
| clean reads | reads/cleaned/ |
| assemblies | assembly/ |
| assembly input | assembly/input/ |
| assembly output | assembly/result/ |
| mappings | mapping/ |
| mapping output | mapping/bams/ |
| annotations | annotations/ |
| annotation input | annotations/input/ |
| annotation output | annotation/features/ |
| error logs | backbone\_errors/ |

Sequence files should be fasta or sanger fastq, fasta for the ones without quality and fastq for the ones with quality. The file extension should reflect the sequence file format, fasta and sfastq.

The sequence file names for the reads should define several tags: lb (library), sm (sample) and pl (platform/technology). This convention is required if a mapping analysis is to be done with this sequence files. These tags follow the sam file header specification. An example of some valid sequence file names is:

```
$ ls reads/raw/
lb_mos.pl_illumina.sm_mos.sfastq  lb_pep.pl_illumina.sm_pep.sfastq
lb_mu16.pl_454.sm_mu16.sfastq     lb_upv196.pl_454.sm_upv196.sfastq
```

# Available analyses¶

The available analyses are:

| analysis | description |
| --- | --- |
| *Cleaning sequence reads* | sequence reads cleaning |
| *Mira assembly* | Assembly reads into a contig set with mira |
| *Mapping* | bwa read mapping against a reference sequence |
| *Bam realignment* | GATK bam realignment |
| *SNP calling* | SNP annotation from a bam file |
| *ORF annotation* | ESTScan ORF annotation |
| *Ortholog annotation* | reciprocal blast based ortholog annotation |
| *Description annotation* | description blast based annotation |
| *Microsatellite annotation* | microsatellite sputnik based annotation |
| *cDNA intron annotation* | est2genome cDNA based annotation |
| *GO annotation* | blast2go annotation |

# Parallel operation¶

Running ngs\_backbone with multiple subprocesses is as easy as setting the configuration option threads to True. ngs\_backbone will run will as many subprocesses as cpu cores are found in the computer. Also the threads option can be set to an integer and ngs\_backbone will run with as many subprocess as indicated.

### Table Of Contents

- Introduction
- Usage
- Naming conventions
- Available analyses
- Parallel operation
- Installation
- Cleaning sequence reads
- Mira assembly
- Mapping
- Bam realignment
- Annotation
- Snv filters
- Tutorials
- NGS workshop
- Licence
- Indices and tables
- seq\_io
- Architecture

### Search


Enter search terms or a module, class or function name.

index |
next |
previous
  
Show Source

© Copyright 2010, Jose Blanca.
Created using Sphinx 1.0pre.
