# Supplementary material for: ngs_backbone: a pipeline for read cleaning, mapping and SNP calling using Next Generation Sequence
Source: BMC Genomics. 2011 Jun 2;12:285. doi: 10.1186/1471-2164-12-285 (PMC3124440; doi:10.1186/1471-2164-12-285)
Supplement: Additional file 1 — ngs_backbone 1.1.0 software. ngs_backbone 1.1.0. Last version, released on 31-08-2010. [file 1471-2164-12-285-S1.GZ › ngs_backbone-1.1.0/doc/annotation.html]

Annotation — ngs\_backbone v0.1 documentation


# ngs\_backbone v0.1 documentation

index |
next |
previous

# Annotation¶

There are different annotation analyses for the sequences, but they all operate in a similar way, so it is worth to explain the general annotation process. The sequences to annotate should be placed in one or several files at annotations/input/. When an annotation is done the results are stored in a kind of database at annotations/db/. There is a versioned file in annotations/db/ for each original file set to annotate. The annotations are cumulative, so if we annotate the ORF and after that the SSRs both will be stored at annotations/db/. At every time after an annotation the real output files can be generated running the write\_annotation analysis. The output files will be found at annotations/features/. The output files are:

- VCF file. It stores the SNP and indel information.
- DNA and pep ORF fasta files. The DNA to translate and the translation.
- a csv file for the SSR (microsatellite) information.
- GFF file. It stores all found sequence features.
- annot and dat files for Blast2Go.
- a text file with the orthologs.

## SNP calling¶

SNP calling is a form of sequence annotation. To annotate some sequences you need the input sequences to annotate, a bam file in mapping/bams/merged.bam and a reference genome in mapping/reference/reference.fasta. SNP calling is based on the samtools pileup with some filters on top. The ngs\_backbone analysis is: annotate\_snv.

### Configuration parameters¶

In the section Snvs:

min\_quality
:   Minimum allele quality. The alleles with a lower quality will not be consider. It is a phred quality.

min\_mapq
:   Reads mapped with a lower quality will not be considered. This is the phred quality found on the bam file.

min\_num\_alleles
:   Usually it will be 1 or 2. If 1 the positions with only one allele different from the reference will be considered if 2 only the positions with at least 2 alleles will be considered.

Also a subsection edge\_removal inside Snvs could be defined. The parameters in this section are used to when we want to ignore the nucleotides close to the edged of the reads. This parameters are dependent on the platform and are: 454\_left, 454\_right, sanger\_left, sanger\_right, illumina\_left and illumina\_right.

## SNV filtering¶

Once you have the snv annotated, you may not want all of them. Only some of then may be of your interest. ngs\_backbone provide an analysis to filter snvs.

Yo can find more information on how to use this analysis *here*.

## Blast databases¶

Several annotations make use of blast. ngs\_backbone requires some data about the blast databases to be able to run. This information should be set up in the ngs\_backbone.conf file on the blast section. In this section for each blast database a subsection for every database should be prepared. An example for the nr would be:

```
[blast]
[[nr]]
path = /absolute/path/to/blast/database
species = 'all'  #this database is not species specific
kind = 'prot'    #or nucl for nucleotide databases
```

## Description annotation¶

A description for the sequences can be created blasting some databases. Several blast databases can be used in a sequential way. Once a sequence has a blast hit in one of the databases, the description will be build from the description of that hit. For instance let’s imagine that we have to annotate 10 sequences with the databases swissprot and tair. Both blasts would be carried out. If we find significant hits for 5 sequences in swissprot those sequence would be already annotated and a description for the 5 remaining sequences will be look for at the second database (tair in this case). The corresponding ngs\_backbone analysis is annotate\_description.

### Configuration parameters¶

In the ngs\_backbone.conf section Annotation, subsection description\_annotation the parameter description\_databases should have a list of at least one blast database. The blast databases used should be defined in the corresponding section, see ::blast-databases.

## GO annotation¶

ngs\_backbone annotates the Gene Ontology ontology terms by using blast2go. Blast2GO uses the results of a blast nr search to infer the relevant GO terms for every sequence. The corresponding blast analysis is annotate\_go.

### Output files¶

The Blast2GO executable creates two files that can be loaded by the graphical Blast2GO interface, the annot and the dat file. Both will be present at the directory annotations/features. Be aware that generating the dat file will require quite memory.

### Configuration parameters¶

In the ngs\_backbone.conf annotation section a go\_annotation subsection should be present. The parameters are:

create\_dat\_file
:   It can be set to True or False

java\_memory
:   This optional parameter is especially important if the creation of the dat file is required.

b2g\_properties\_file
:   Optionaly you can configure blast2go’s properties tunning this file.

## ORF annotation¶

To annotate the ORFs found in your sequences just run the ngs\_backbone analysis annotate\_orf. ESTScan will be used to look for the ORFs. The output will be (after running write\_annotation) a couple of files for each input file, one for the DNA and another for the proteins.

### Configuration parameters¶

In order to run this analysis in the section orf\_annotation at the ngs\_backbone.conf file the estscan\_matrix matrix file should be defined. This is a valid specific matrix file for ESTScan

## Microsatellite annotation¶

The SSRs can be annotated just by running the ngs\_backbone analysis annotate\_microsatellite. The result of this analysis will be shown in the gff file and in a csv microsatellite file.

## Ortholog annotation¶

ngs\_backbone can annotate the orthologs doing a reciprocal blast search. It can be done on one or several blast databases. The ngs\_backbone analysis is called ortholog\_annotation. The list of orthologs will be found in annotations/features/

### Configuration parameters¶

In the ngs\_backbone.conf section Annotation, subsection ortholog\_annotation the parameter ortholog\_databases should have a list of at least one blast database. The blast databases used should be defined in the corresponding section, see ::blast-databases.

## cDNA intron annotation¶

When the sequences to annotate are cDNA ngs\_backbone can guess where the introns were by using the analysis annotate\_introns. To do it it aligns the cDNA with a genomic sequence using the emboss program est2genome. As a shortcut ngs\_backbone before running est2genome with the whole genomic sequence it does a blast search to look for the relevant genome region and only after that the est2genome alignment is done.

### Configuration parameters¶

In the ngs\_backbone.conf section Annotation, subsection Cdna\_intron\_annotation the parameter genomic\_db should have one blast database. The blast database used should be defined in the corresponding section, see ::blast-databases. Also in the same section the parameter genomic\_seqs should have the absolute path to the fasta file with the genomic sequences that make up the employed database.

## Annotation statistics¶

Once we have annotated some sequences we can get a summary of the process by running the annotation\_stats analysis. It will create one text file for each input annotation file with statistics about: description, microsatellites, SNVs, ORF, GO terms and orhologs.

### Table Of Contents

- Introduction
- Usage
- Naming conventions
- Available analyses
- Parallel operation
- Installation
- Cleaning sequence reads
- Mira assembly
- Mapping
- Bam realignment
- Annotation
  - SNP calling
  - SNV filtering
  - Blast databases
  - Description annotation
  - GO annotation
  - ORF annotation
  - Microsatellite annotation
  - Ortholog annotation
  - cDNA intron annotation
  - Annotation statistics
- Snv filters
- Tutorials
- NGS workshop
- Licence
- Indices and tables
- seq\_io
- Architecture

### Search


Enter search terms or a module, class or function name.

index |
next |
previous
  
Show Source

© Copyright 2010, Jose Blanca.
Created using Sphinx 1.0pre.
