# Supplementary material for: ngs_backbone: a pipeline for read cleaning, mapping and SNP calling using Next Generation Sequence
Source: BMC Genomics. 2011 Jun 2;12:285. doi: 10.1186/1471-2164-12-285 (PMC3124440; doi:10.1186/1471-2164-12-285)
Supplement: Additional file 1 — ngs_backbone 1.1.0 software. ngs_backbone 1.1.0. Last version, released on 31-08-2010. [file 1471-2164-12-285-S1.GZ › ngs_backbone-1.1.0/doc/seqio.html]

seq\_io — ngs\_backbone v0.1 documentation


# ngs\_backbone v0.1 documentation

index |
next |
previous

# seq\_io¶

seq\_io.py is a little utility distributed with ngs\_backbone that allows us to move sequence files from one sequence format to other. To change between formats we just have to tell seq\_io.py which are the input and output files and which are the input and output formats. The parameters to use are:

```
$ seqio.py -h
Usage: seqio.py [options]

Options:
  -h, --help            show this help message and exit
  -s INSEQFILE, --inseqfile=INSEQFILE input sequence file
  -q INQUALFILE, --inqualfile=INQUALFILE input quality file
  -f INFORMAT, --informat=INFORMAT input file format
  -o OUTSEQFILE, --outseqfile=OUTSEQFILE output sequence file
  -l OUTQUALFILE, --outqualfile=OUTQUALFILE output quality file
  -t OUTFORMAT, --outformat=OUTFORMAT output file format
```

For instance, to go from sanger fastq to fasta and qual we would do:

```
$ seqio.py -s seq.sfastq -f sfastq -o seq.fasta -l seq.qual -t fasta
```

And to do the reverse:

```
$ seqio.py -s seq.fasta -q seq.qual -f fasta -o seq.sfastq -t sfastq
```

### Table Of Contents

- Introduction
- Usage
- Naming conventions
- Available analyses
- Parallel operation
- Installation
- Cleaning sequence reads
- Mira assembly
- Mapping
- Bam realignment
- Annotation
- Snv filters
- Tutorials
- NGS workshop
- Licence
- Indices and tables
- seq\_io
- Architecture

### Search


Enter search terms or a module, class or function name.

index |
next |
previous
  
Show Source

© Copyright 2010, Jose Blanca.
Created using Sphinx 1.0pre.
