# Supplementary material for: ngs_backbone: a pipeline for read cleaning, mapping and SNP calling using Next Generation Sequence
Source: BMC Genomics. 2011 Jun 2;12:285. doi: 10.1186/1471-2164-12-285 (PMC3124440; doi:10.1186/1471-2164-12-285)
Supplement: Additional file 1 — ngs_backbone 1.1.0 software. ngs_backbone 1.1.0. Last version, released on 31-08-2010. [file 1471-2164-12-285-S1.GZ › ngs_backbone-1.1.0/doc/cleaning.html]

Cleaning sequence reads — ngs\_backbone v0.1 documentation


# ngs\_backbone v0.1 documentation

index |
next |
previous

# Cleaning sequence reads¶

ngs\_backbone can clean sanger, 454 and illumina sequences. This process usually involves vector and adaptor removal, bad quality regions trimming and short sequence filtering. There are three cleaning pipelines defined in ngs\_backbone that are used depending on the platform and on the quality availability:

long reads with quality
:   for sanger and 454 sequences with quality information

long reads without quality
:   for sanger reads without quality information

solexa
:   for short illumina reads

A collection of cleaning steps are available that compose each one of these pipelines. These steps are:

adaptor removal
:   Each sequence is align against the adaptors found in a fasta file. The external tool used to do the matching is exonerate. If a match is found this section of the read is removed. Short adaptors will be treated as such.

short adaptor removal
:   ngs\_backbone will look for adaptors shorter than 15 bp with exact matches.

precise vector removal
:   If the vector and cloning site is known lucy can be used to remove the vector in a precise way.

bad quality trimming
:   There are two algorithms used to remove the bad quality sequence extremes. If the sequence is long lucy (454 and sanger) is used for this task otherwise ngs\_backbone does the job (illumina).

bad quality trimming by Ns
:   When there are no qualities available we can infer the quality from the percent of Ns found in the sequence. This step removes regions with a high number of Ns.

general vector removal
:   The reads are compared against the Univec database using blast to look for remaining vectors.

low complexity masking
:   The regions with a low complexity are masked by using mdust

edge removal
:   After all the other modules are run we can delete a fixed amount of bases from the sequence extremes

short sequence filtering
:   When the process for one sequence is completed a minimum length criteria is applied.

The pipelines are:

long reads with quality
:   adaptor removal, precise vector removal, bad quality trimming, general vector removal, low complexity masking, word removal, edge removal, and short sequence filtering

long reads without quality
:   general vector removal, bad quality trimming by Ns, low complexity masking, word removal, edge removal, and short sequence filtering

solexa
:   adaptor removal, bad quality trimming, and short sequence filtering

## Input and output files¶

The reads to be cleaned should be in the project directory under /reads/raw/. The *naming conventions* should be followed by these files, especially the bit regarding to the extension. The output files will have the same names, but they will be located at /reads/cleaned/. The analysis will proceed for all sequence files found in /reads/raw, if a matching file is not found in /reads/cleaned/ a new cleaned file will be generated. If a matching file is found in /reads/cleaned/ these file will not be overwritten, so the analysis for this file will not be repeated until the file from /reads/cleaned is removed.

## Configuration parameters¶

The configuration for the cleaning analysis is found in the Cleaning section on the ngs\_backbone.conf file. The parameters are:

vector\_database
:   The blast database that will be used to look for clonning vectors.

adaptors\_file\_454
:   A path to a fasta file containing the adaptors used to build the 454 library. They will be removed from the cleanend reads.

adaptors\_file\_sanger
:   Idem for the sanger sequences

adaptors\_file\_illumina
:   Idem for the illumina sequences

short\_adaptors\_454
:   A list of words to be removed. They can be regular expressions.

short\_adaptors\_sanger
:   Idem for the sanger sequences

short\_adaptors\_illumina
:   Idem for the illumina sequences

edge\_removal -> 454\_left
:   A fixed number of bases to be removed from the left edge of the 454 reads.

edge\_removal -> 454\_right
:   Idem for the right edge of the 454 reads

edge\_removal -> sanger\_left
:   Idem for the left edge of the sanger reads

edge\_removal -> sanger\_right
:   Idem for the right edge of the sanger reads

edge\_removal -> illumina\_left
:   Idem for the left edge of the illumina reads

edge\_removal -> illumina\_right
:   Idem for the right edge of the illumina reads

strip\_n\_percent
:   Threshold used for the trimming of regions with a lot of Ns in sequences with no quality information available. Lower values (e.g. 1.5) are more stringent.

min\_seq\_length
:   The minimum sequence length allowable after the cleaning is done. All sequences shorter than these values will be discarded. This is a subsection with one value for each platform 454, sanger and illumina.

lucy -> vector\_settings
:   A path to a lucy settings file with the splice and vectors files to be used by lucy

lucy ->bracket
:   Look at lucy man page before changing defaults.

lucy -> window
:   Look at lucy man page before changing defaults.

lucy -> error
:   Look at lucy man page before changing defaults.

## lucy settings¶

The lucy settings file should have the following format:

```
{'library1':{'vector_file':'lib1_vector.fasta', 'splice_file':'lib1_splice.fasta'},
 'library2':{'vector_file':'lib2_vector.fasta', 'splice_file':'lib2_splice.fasta'},}
```

In this file the paths to the vector and splice files for lucy should be stated for every library to be cleaned by lucy. The library name will be scraped from the read sequence file (that should follow the *naming conventions*. The vector file is just a fasta file, the information to be set in the splice file should is explained in the lucy man page.

### Table Of Contents

- Introduction
- Usage
- Naming conventions
- Available analyses
- Parallel operation
- Installation
- Cleaning sequence reads
  - Input and output files
  - Configuration parameters
  - lucy settings
- Mira assembly
- Mapping
- Bam realignment
- Annotation
- Snv filters
- Tutorials
- NGS workshop
- Licence
- Indices and tables
- seq\_io
- Architecture

### Search


Enter search terms or a module, class or function name.

index |
next |
previous
  
Show Source

© Copyright 2010, Jose Blanca.
Created using Sphinx 1.0pre.
