# Supplementary material for: ngs_backbone: a pipeline for read cleaning, mapping and SNP calling using Next Generation Sequence
Source: BMC Genomics. 2011 Jun 2;12:285. doi: 10.1186/1471-2164-12-285 (PMC3124440; doi:10.1186/1471-2164-12-285)
Supplement: Additional file 2 — Materials and methods. materials and methods, for the experimental software validation. [file 1471-2164-12-285-S2.PDF]

Materials and Methods

DNA and plant material

The tomato collection used to evaluate SNV polymorphism was composed of 27 tomato landraces provided by the COMAV genebank and ten commercial cultivars grown thoughout Spain (Additional file 4). DNAs were isolated from fresh young leaves using the CTAB protocol (Dellaporta et al, 1983).

cDNA library Illumina sequencing

Total RNA from 250mg of flowers at the anthesis stage of UC82 and RP75/59 plants were extracted with TRI Reagent (Sigma-Aldrich, Saint Louis, USA) following the manufacturer's instructions. Both total RNAs were mixed at equimolar concentrations and the mix was purified with the mRNA Purification kit (Amersham Biosciencies, Buckinghamshire, UK). 1 μg mRNA sample was used for the cDNA synthesis with Clontech's SMART cDNA Synthesis kit (Clontech, Mountain View, USA). The cDNA library was normalized with the Trimmer kit (Evrogen, Moscow, Rusia) and sequenced on a 1/8 Illumina run.

Genotyping

The PCR primers used to amplify the SNV-containing regions were designed with the PrimerExpress software (Applied Biosystems, Carlsbad, CA, USA). HRM PCR reactions (Gubdry et al, 2003) were carried out in a LC480 Roche PCR (Roche Basel, Schweiz) employing the LC480 High Resolution Melting master mix kit (Roche Basel, Schweiz). UC-82 and RP75/79 tomato varieties as well as the F1 hybrid developed from them were used as HRM PCR controls to calibrate the HRM parameters and to identify the HRM profiles with the different alleles. The cases in which only the heterozygous were distinguished by HRM were analyzed by adding DNA from a homozygous control to the tested sample.

The PCR primers used are shown in Additional file 5.

The CAPs were tested in a 2% agarose gel after digesting the PCR product with the required restriction enzyme predicted *in silico*.

References

SL Dellaporta, J Wood, JB Hicks: **A plant DNA minipreparation: version II**. In., vol. 1. Plant Mol Biol Rep; 1983: 19-21.

Gundry CN, Vandersteen JG, Reed GH, Pryor RJ, Chen J, Wittwer CT: **Amplicon melting analysis with labeled primers: A closed-tube method for differentiating homozygotes and heterozygotes**. *Clinical Chemistry* 2003, **49**(3):396-406.
